# Integrated analysis of circulating immune cellular and soluble mediators reveals specific COVID19 signatures at hospital admission with utility for prediction of clinical outcomes

**DOI:** 10.7150/thno.63463

**Published:** 2022-01-01

**Authors:** Iratxe Uranga-Murillo, Elena Morte, Sandra Hidalgo, Cecilia Pesini, Sandra García-Mulero, Jose L. Sierra, Llipsy Santiago, Maykel Arias, Diego De Miguel, María del Mar Encabo-Berzosa, Borja Gracia-Tello, Rebeca Sanz-Pamplona, Luis Martinez-Lostao, Eva M. Galvez, Jose R. Paño-Pardo, Ariel Ramirez-Labrada, Julian Pardo

**Affiliations:** 1 Fundación Instituto de Investigación Sanitaria Aragón (IIS-Aragón), Biomedical Research Centre of Aragon (CIBA), 50009 Zaragoza, Spain.; 2 Servicio de Enfermedades Infecciosas, Hospital Clinico Universitario Lozano Blesa, 50009 Zaragoza, Spain.; 3 Unit of Biomarkers and Susceptibility, Oncology Data Analytics Program (ODAP), Catalan Institute of Oncology (ICO), Oncobell Program, Bellvitge Biomedical Research Institute (IDIBELL) and CIBERESP, L'Hospitalet de Llobregat, Barcelona, Spain.; 4 Department of Immunology, University Clinic Hospital Lozano Blesa, 50009, Zaragoza, Spain and Department of Pathology, University Clinic Hospital Lozano Blesa, University of Zaragoza, IIS Aragón, 50009 Zaragoza, Spain.; 5 Instituto de Carboquímica ICB-CSIC, 50018 Zaragoza, Spain.; 6 Biobanco de Aragón, Instituto Aragonés de Ciencias de la Salud (IACS), 50009, Zaragoza, Spain.; 7 Servicio de Enfermedades Infecciosas, Hospital Clinico Universitario Lozano Blesa, 50009 Zaragoza, Spain.; 8 Nanoscience Institute of Aragon (INA), Consejo Superior de Investigaciones Científicas (CSIC), University of Zaragoza, 50018 Zaragoza, Spain.; 9 Department Microbiology, Pediatry, Radiology and Public Health, University of Zaragoza.; 10 Unidad de nanotoxicología e inmunotoxicología experimental (UNATI). Fundación Instituto de Investigación Sanitaria Aragón (IIS-Aragón), Biomedical Research Centre of Aragón (CIBA), 50009 Zaragoza, Spain.; 11 Centro de Investigación Biomédica en Red (CIBER) de Enfermedades Infecciosas, 50018 Madrid, Spain.; 12 Aragon I+D Foundation (ARAID), Zaragoza, Spain.

**Keywords:** COVID19, GzmA, GzmB, NK cells, CXCL9, CXCL10, MIC, ULBP

## Abstract

Coronavirus disease 2019 (COVID19), caused by SARS-CoV-2, is a complex disease, with a variety of clinical manifestations ranging from asymptomatic infection or mild cold-like symptoms to more severe cases requiring hospitalization and critical care. The most severe presentations seem to be related with a delayed, deregulated immune response leading to exacerbated inflammation and organ damage with close similarities to sepsis.

**Methods:** In order to improve the understanding on the relation between host immune response and disease course, we have studied the differences in the cellular (monocytes, CD8+ T and NK cells) and soluble (cytokines, chemokines and immunoregulatory ligands) immune response in blood between Healthy Donors (HD), COVID19 and a group of patients with non-COVID19 respiratory tract infections (NON-COV-RTI). In addition, the immune response profile has been analyzed in COVID19 patients according to disease severity.

**Results:** In comparison to HDs and patients with NON-COV-RTI, COVID19 patients show a heterogeneous immune response with the presence of both activated and exhausted CD8+ T and NK cells characterised by the expression of the immune checkpoint LAG3 and the presence of the adaptive NK cell subset. An increased frequency of adaptive NK cells and a reduction of NK cells expressing the activating receptors NKp30 and NKp46 correlated with disease severity. Although both activated and exhausted NK cells expressing LAG3 were increased in moderate/severe cases, unsupervised cell clustering analyses revealed a more complex scenario with single NK cells expressing more than one immune checkpoint (PD1, TIM3 and/or LAG3). A general increased level of inflammatory cytokines and chemokines was found in COVID19 patients, some of which like IL18, IL1RA, IL36B and IL31, IL2, IFNα and TNFα, CXCL10, CCL2 and CCL8 were able to differentiate between COVID19 and NON-COV-RTI and correlated with bad prognosis (IL2, TNFα, IL1RA, CCL2, CXCL10 and CXCL9). Notably, we found that soluble NKG2D ligands from the MIC and ULBPs families were increased in COVID19 compared to NON-COV-RTI and correlated with disease severity.

**Conclusions:** Our results provide a detailed comprehensive analysis of the presence of activated and exhausted CD8+T, NK and monocyte cell subsets as well as extracellular inflammatory factors beyond cytokines/chemokines, specifically associated to COVID19. Importantly, multivariate analysis including clinical, demographical and immunological experimental variables have allowed us to reveal specific immune signatures to i) differentiate COVID19 from other infections and ii) predict disease severity and the risk of death.

## Introduction

Since December 2019 and up until October 2021, Severe Acute Respiratory Syndrome Coronavirus type 2 (SARS-CoV-2), the causative agent of the coronavirus disease 2019 (COVID19) pandemic, has infected more than 230 million people, causing more than 4.7 million deaths 1. SARS-CoV-2 infects epithelial cells from the upper respiratory tract and in most cases patient immune response will efficiently control infection, remaining asymptomatic or with mild cold-like symptoms. If virus avoids host immunity in the upper respiratory tract, it will colonise the lower respiratory tract where it can cause a more serious disease including pneumonia and in some cases death. Here the virus infects alveolar type II (AT2) cells 2 that comprise 15% of total lung tissue and maintain the alveolar microenvironment function 3. The airway epithelial cells (AECs) provide both physical and immunological barriers to protect from pathogens invading the respiratory tract. AECs express recognition receptors (PRRs) to rapidly detect and respond to pathogen-associated molecular patterns (PAMPs), which leads to the release of cytokines, chemokines and antimicrobial peptides that attract and activate immune cells 4. The activation of an efficient regulated immune response is key for a successful control of respiratory viral infections, before pathogen increases progeny and triggers an exacerbated deregulated immune response leading to self-tissue damage and disease. A good example of the dichotomy infection control versus immunopathology is represented by SARS coronaviruses. SARS-CoV-2 seems to have a dual nature, tragically lethal in some people and surprisingly benign in others. Although the reasons for these interpersonal differences are currently unknown it has been suggested that the generation of a strong innate immune response capable of restricting viral replication and/or the presence of some memory due to a previous infection with seasonal common cold coronaviruses which restrict virus replication might avoid severe infections 5-7. Indeed, mild cases and good recovery within hospitalized patients have been related to an early and robust response mediated by activated virus-specific CD8^+^ and CD4^+^ T cells 8. An initial inefficient innate and adaptive immune response due to aging and/or other medical conditions might lay behind severe infections 9, 10. The delayed immune responses might generate an exacerbated imbalanced response, characterized by strong inflammation associated to over-production of pro-inflammatory cytokines and deregulation of lymphocytes including a reduction in number and functionality 11-16. In addition, markers of T and NK cell exhaustion have been found during COVID19, which is enhanced in severe cases, albeit the relevance of activated and exhausted cell populations in COVID19 is not completely understood yet 13, 17-20. Despite these studies, there are still many caveats to properly understand the regulation of host immunity during SARS-CoV-2 infection and importantly, to reveal its potential utility to monitor clinical evolution and predict disease severity. Specially, since most studies have compared the immune response in COVID19 with healthy donors, without including patient cohorts with other infections, which does not allow to reveal if there is an immune response profile specifically associated to SARS-CoV-2 infection. A major understanding of these responses will allow developing rational treatments to tune up the immune response in COVID19, promoting viral clearance and/or preventing immunopathology, without affecting the host protective immune response.

Here we have performed a prospective multiparametric study of the immune response in blood from 86 COVID19 patients collected at hospital admission and compared with 40 healthy donors and 27 patients with other respiratory infections, focusing in some of the main cells involved in viral immunity (monocytes, CD8^+^T and NK cells) and in different families of soluble factors regulating their function (cytokines, chemokines, granzymes/Gzms and soluble NK cell ligands). The main objective of this work is to characterize COVID19 patient's immunological profile at hospitalisation to better understand the regulation of host immunity in response to SARS-CoV-2 infection and to find out if immune profiling presents utility to differentiate COVID19 from other infections and to establish patient prognosis.

## Methods

### Patients, clinical data collection and sample processing

Patients admitted to the "Lozano Blesa" University Hospital of Zaragoza with suspected SARS-CoV-2 infection were screened for SARS-CoV-2 (Prospective study from April to May 2020). Healthy donors (HDs) were adults with no prior diagnosis or symptoms consistent with COVID19 recruited in April 2020. COVID19 was confirmed by PCR and/or serological test. The patients who presented symptoms compatible with COVID19 but diagnosis was not confirmed by PCR nor serological test were classified as patients with non-COVID19 respiratory tract infections (NON-COV-RTI). HDs meeting standard normal donor eligibility criteria were recruited to donate blood. Eligibility criteria for voluntary whole blood donation are: healthy, male or female from 18 to 65 years old with a weight > 50 Kg, without history of heart, lung, kidney disease, chronic anaemia or bleeding disorders and negative to COVID19, VIH, hepatitis B virus, hepatitis C virus, syphilis, HTLV1 and 2. The investigational nature of the studies in which their samples will be used, as well as the risks and benefits of the donation process was explained to all donors, and a signed informed consent document was obtained. All samples (HD and patients) were collected by the Aragon Biobank which is accredited by the Aragon Government and the study was approved by the local ethics committee (CEICA) which is accredited by the Spanish Agency for Medicaments and Sanitary products (AEMPS). The time from sample collection to analyses was 24-48h in all cases. The final sample size was n = 48, n = 86 and n = 27 for HDs, COVID19 and NON-COV-RTI patients respectively. The latter is a heterogeneous respiratory infection patient cohort with clinical pictures similar to that one of COVID19 patients that would allow us to detect the specific immune response profile associated to COVID19 in a real life situation. Samples and data from patients included in this study were provided by the Biobank of the Aragon Health System, integrated in the Spanish National Biobanks Network and they were processed following standard operating procedures with the appropriate approval of the Ethics and Scientific Committee (CEIC Aragon, number PI20/165). Peripheral blood was collected from all participants within the first 24h after hospital admission before any treatment was prescribed and clinical data were compiled from the electronic medical record into standardized case report forms. A Flow Diagram of the progress of the trial has been included as Figure S1. Serum, plasma and peripheral blood mononuclear cells (PBMCs) were obtained as described in supplementary materials.

### Flow cytometry and unsupervised analysis

PBMCs were stained with specific antibodies using 4 different panels. Panel 1 (T^reg^ cells): CD3, CD4, CD127, CD25. Panel 2 (activating/inhibitory NK cell receptors): CD3, CD56, CD16, CD57, NKG2A, NKG2C, NKG2D, NKp30, NKp46. Panel 3 (activated/exhausted NK cells and Monocytes): CD3, CD56, CD16, CD14, GzmB, TIM3, LAG3, PD1, HLA-DR. Panel 4 (Activated/Exhausted CD8+T cells):CD3, CD8, GzmB, TIM3, LAG3, PD1, CD38, HLA-DR. A detailed list of antibodies and conventional and unsupervised flow cytometry data analysis are indicated in supplementary materials. The gating strategy followed for cell population and marker expression analysis is shown in Figure S2.

### Interferon-γ (IFN-γ) expression assay

A functional study of the interferon-γ (IFN-γ) expression by T cells was performed. PBMCs were stimulated with a SARS-CoV-2 peptide mixture (S, M and N proteins) for 6 hours, at 37 ºC, 5 % CO_2_. Besides, PBMCs were stimulated with CytoStim (Milteny) as positive control and without stimulation as negative controls. To detect IFN-γ secretion by T cells the IFN-γ Secretion Assay-Cell Enrichment and Detection Kit (PE) for humans (Miltenyi) was used. Briefly, each sample was pre-incubated with 4 µl of IFN-γ Catch Reagent, 5 min at 4 ºC, and after dilution with complete medium was incubated for 45 min, 37 ºC and rotation. PBMCs were washed and stained with 4 µl of IFN-γ Detection Antibody PE, for detection of secreted IFN-γ, and 5 µl of Rapid Cytokine Inspector in order to define the T cell populations (CD3+, CD4+, CD8+). This mixture was then incubated for 15 min at 4 ºC and darkness, and finally all samples were fixed with 2 % PFA and analysed by flow cytometry.

### Multiplex plasma protein analyses and enzyme activity assays

Luminex assay was run according to manufacturer's instructions in 100 µl of plasma, using a custom human cytokine panel (R&D Systems, catalogue no. LXSAHM). A detailed list of the proteins included and their analysis is indicated in supplementary materials. Granzyme A and B activity was analysed in serum using specific quenching FRET fluorescent substrates (FAM-VANRSAS-DABCYL and FAM-IEPDNLV-DABCYL peptides, respectively) as described in supplementary materials.

### Statistics

A detailed description of univariate and multivariate statistical analysis is included in supplementary materials.

## Results

### Demographical and clinical variables of COVID19 and NON-COV-RTI patients

113 consecutive patients with respiratory infection symptoms compatible with COVID19 were included. 86 patients were confirmed as SARS-CoV-2 infection (COVID19) and the other 27 patients, presenting COVID19-like symptoms but negative RT-PCR and serologic testing, were classified as NON-COV-RTI, since specific diagnosis was not accomplished. A summary of the demographical and clinical variables according to diagnosis and severity is shown in Tables S1 and S2. Sex, age, number of days from symptoms onset to blood sampling and median length of hospital stay were not significantly different between the cohorts Table S1. Lymphocyte count was significantly lower in COVID19 than in NON-COV-RTI. Similarly to the different diagnosis groups, number of days from symptom onset to sampling was not significantly different between mild and moderate/severe COVID19 Table S2. The classification in mild and moderate/severe cases agrees with known factors about COVID19 severity, as age. In fact, median age, but not gender, was significantly higher in moderate/severe cases (77.9) than in mild cases (61). When patients were grouped in mild/moderate and severe cases, there were no significant differences in age (75 and 80 respectively). The moderate/severe group presented significant lower lymphocyte counts (794 cell/µL) than the mild cases (1225 cell/µL) and significantly longer hospital stay (mean of 17 and 6 days respectively) further supporting the severity classification Table S2. Regarding comorbidities, COVID19 severity was significantly increased in patients with chronic heart and neurological disease, hypertension, Diabetes mellitus, obesity and dementia Table S3. Comorbidities were also compared between COVID19 and NON-COV-RTI patients with no significant differences Table S4.

### CD8^+^T, NK and Monocyte cell profiles in COVID19, NON-COV-RTI and healthy donors

**Figure 1:**
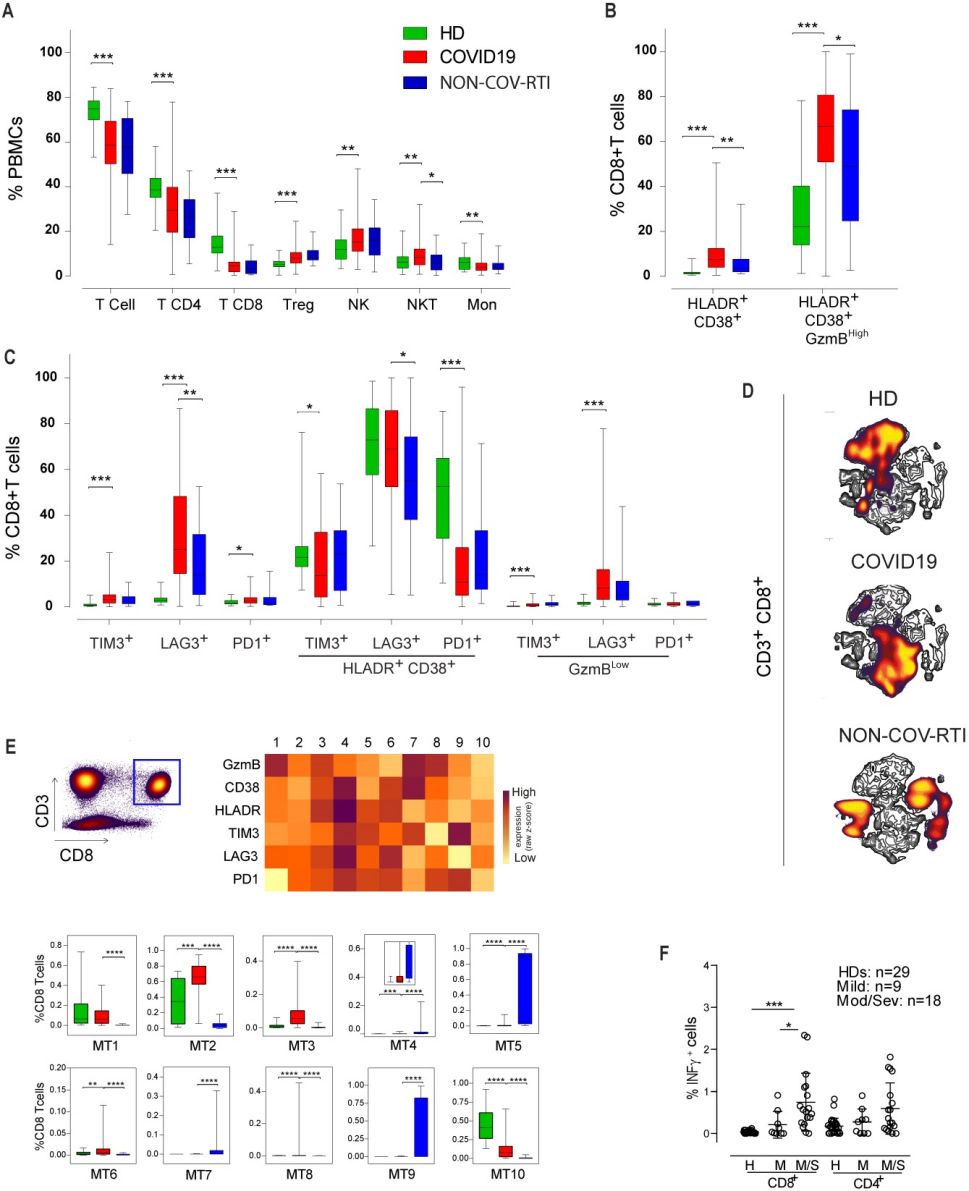
Analysis of the main immune cell populations and activated and exhausted CD8^+^T cell subsets in blood from COVID19 patients compared with healthy donors (HD) and NON-COV-RTI patients. The presence of different immune cell populations was analysed in PBMCs by flow cytometry as indicated in methods. **A)** Frequencies of total T, CD4^+^T, CD8^+^T, T^reg^, NKT and NK cells and monocytes. **B) C)** Frequencies of activated and exhausted CD8^+^T cells. **D)** viSNE maps of CD8^+^T cells from COVID19, HD and NON-COV-RTI. **E)** Mean fluorescence intensity (MFI) in each FlowSOM metacluster (z-scores) of GzmB, CD38, HLA-DR, TIM3, LAG3 and PD1 on CD8^+^T cells and percentage of CD8^+^T cells in each FlowSOM metacluster. Boxes represent interquartile ranges (IQRs). Statistical significance was determined by unpaired Mann-Whitney or Kruskal-Wallis tests as indicated in methods: *p < 0.05, **p < 0.01, ***p < 0.001 and ****p < 0.0001. F) Analyses of SARS-CoV-2 specific T cell functional responses by IFN-γ secretion assay. PBMCs from some COVID19 (n = 27) and HDs (n = 29) were stimulated with a mixture of S, M and N derived peptides (Peptivator, Miltenyi) for 6h and IFN-γ secretion was analysed in CD4+T and CD8+T cells by flow cytometry as indicated in methods. Statistical significance was determined by unpaired Mann-Whitney or Kruskal-Wallis test: as indicated in methods: *p < 0.05, **p < 0.01, ***p < 0.001 and ****p < 0.0001.

In order to analyse the immune response against SARS-CoV-2 infection, first we compared the major PBMC populations between COVID19 patients and healthy donors (Figure 1A). We observed a decrease in CD3^+^ T cell frequency, in both CD4^+^ and CD8^+^ subpopulations, in COVID19 patients compared with HDs reflecting clinical lymphopenia, and an increase in the CD4^+^ regulatory T (T^reg^) cell population. We also found an increase in the frequency of both NKT and NK cells while monocyte frequency decreased (Figure 1A). When COVID19 patients were compared with NON-COV-RTI significant differences were only observed in an increase of NKT cells in COVID19 patients.

The expression of both activation (CD38^+^HLA-DR^+^GzmB^High^; Figure 1B) and exhaustion (TIM3^+^, LAG3^+^, PD1^+^; Figure 1C) markers in CD8^+^T cell population were significantly increased in COVID19 comparing with HD (Figure 1B/C). However, when Immune Checkpoint (IC) expression was analysed separately in either activated (HLA-DR^+^CD38^+^) or exhausted (GzmB^Low^) CD8^+^ T cells, it was found that the frequency of activated CD8^+^ T cells expressing either TIM3 or PD1 was significantly reduced, meanwhile the frequency of exhausted T cells expressing either TIM3 or LAG3 was increased. An increase in activated CD8^+^ T cells is also observed in COVID19 patients in comparison with NON-COV-RTI (Figure 1B), although the frequency of exhausted CD8^+^ T cells (GzmB^Low^) did not change (Figure 1C). The viSNE maps generated by unsupervised flow cytometry analysis for COVID19, NON-COV-RTI and HDs show several distinct partial regions reflecting CD8^+^ T cell heterogeneity (Figure 1D). Striking differences in densities of particular localised regions were found, implying altered relative abundances of CD8^+^T cell subpopulations between COVID19 patients, NON-COV-RTI and HDs that differentially identified each cohort. Minimum Spanning Tree (MST) generated by FlowSOM also showed clear differences between patients and HDs Figure S3A), identifying ten major CD8^+^ T cell metaclusters (MTs) based on GzmB, CD38, HLA-DR, TIM3, LAG3 and PD1 expression (Figure 1E). MT2, 3, 6 and 8 were increased in COVID19 patients with respect to HDs and NON-COV-RTI (Figure 1E). MT3 and MT8 reflects activated CD8^+^T cells (CD38^+^HLA-DR^+^GzmB^High^) expressing all IC or LAG3 and PD1 respectively, while MT2 and MT6 reflect exhausted CD8^+^T cells expressing all IC (CD38^+^HLA-DR^+^GzmB^Low^IC^+^). A functional study of the interferon-γ (IFN-γ) secretion by T cells stimulated with a mixture of SARS-CoV-2 derived peptides (S, M and N) show a significant difference in the CD8+T cell production of IFN-γ between HD and moderate/severe COVID19 as well as between mild and moderate/severe COVID19 patients (Figure 1F).

**Figure 2:**
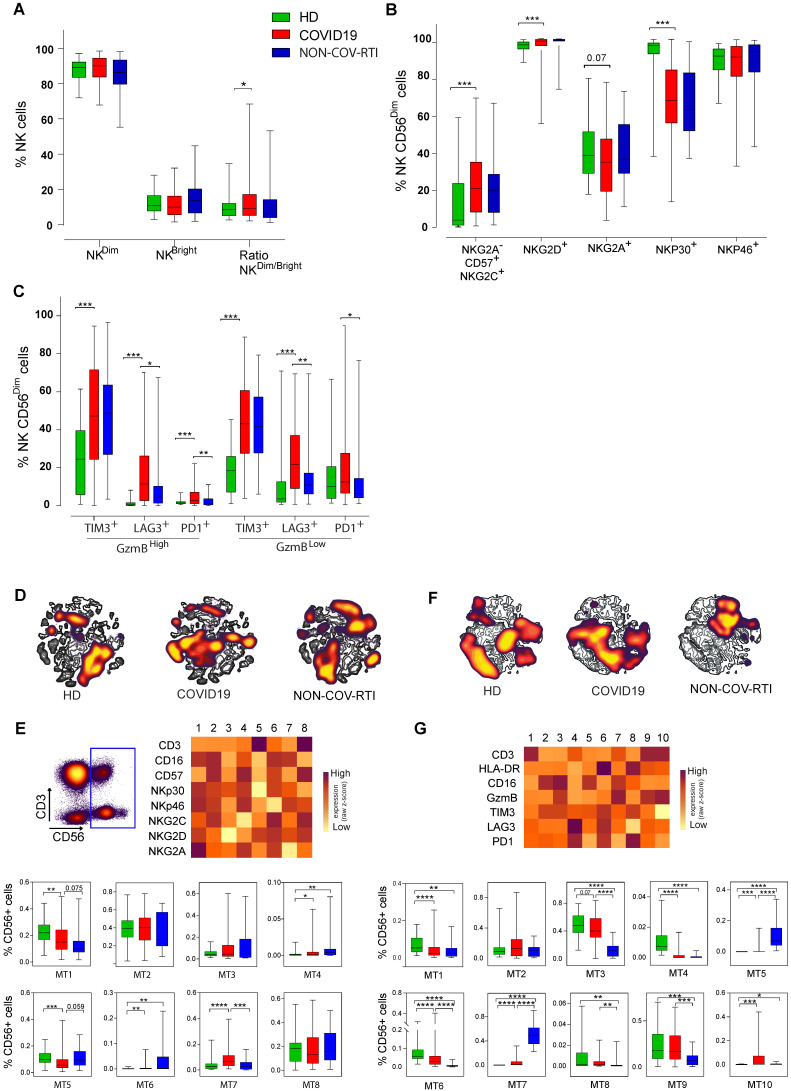
Analysis of activated and exhausted NK cell profiles in blood from COVID19 patients compared with HD and NON-COV-RTI. The presence of different immune cell populations was analysed in PBMCs by flow cytometry as indicated in methods. **A)** Frequencies of the two major NK cell subsets, NK^CD56Dim^ and NK^CD56Bright^ cells. **B)** Expression of activation and inhibitory receptors in NK cells; C-type lectin receptors (NKG2C, NKG2D and NKG2A) and natural cytotoxicity receptors (NCRs) (NKp30 and NKp46) in the NK^CD56Dim^ cell subset. **C)** Expression of activation and exhaustion markers in NK^ CD56Dim^ subset, **D, F)** viSNE maps of CD56^+^ NK cell populations from COVID19 patients, HD and NON-COV-RTI. **E, G)** Heat maps showing the Mean fluorescence intensity (MFI) in each FlowSOM metacluster (z-scores) of activating/inhibitory receptors (E: CD3, CD16, CD57, NKp30, NKp46, NKG2C, NKG2D and NKG2A) or activation/exhaustion markers (F: CD3, HLA-DR, CD16, GzmB, TIM3, LAG3 and PD1) in CD56^+^ NK cells. The percentage of CD56^+^ cells in each FlowSOM metacluster is shown in the graphs. Boxes represent interquartile ranges (IQRs). Statistical significance was determined by unpaired Mann-Whitney or Kruskal-Wallis test: as indicated in methods: *p < 0.05, **p < 0.01, ***p < 0.001 and ****p < 0.0001.

Significant differences in the immunoregulatory (NK^CD56BrightCD16Low^)/cytotoxic (NK^CD56DimCD16Bright^) ratio between COVID19 and HD were found (Figure 2A), although differences in individual subsets were not detected. The expression of activation (NKp30/46, NKG2C/D, CD16), differentiation (CD57) and inhibitory (NKG2A) receptors in the NK^CD56Dim^ cell subset is shown in Figure 2B. The adaptive NK cell subpopulation (CD16^+^NKG2C^+^CD57^+^NKG2A^-^) showed a marked increase in COVID19 versus HD, reaching a median of 21%. A significant increase and a significant decrease were found in NK cells expressing the activation receptors NKG2D and NKp30, respectively. No differences were found between COVID19 and NON-COV-RTI. Similarly, to the main population of CD8^+^ T cells and to exhausted CD8^+^ T cells, we found a significant increase in activated (GzmB^High^PD1^+^, GzmB^High^LAG3^+^, GzmB^High^TIM3^+^) and exhausted (GzmB^Low^PD1^+^, GzmB^Low^LAG3^+^, GzmB^Low^TIM3^+^) NK^CD56Dim^ cells in COVID19 in comparison with HD and with NON-COV-RTI (Figure 2C). Although differences on individual IC were found, notably only LAG3^+^ activated and exhausted cells were consistently increased in COVID19. Notably, the frequency of exhausted NK cells expressing LAG3 (21.6%) or PD1 (12.5%) are higher than their homologous activated NK cells (LAG3, 11.4%; PD1, 2.9%), suggesting a predominance of exhausted NK cells in COVID19 (Figure 2C). viSNE representation revealed a distinctive picture of NK cell subpopulations in COVID19 patients compared with HDs and NON-COV-RTI (Figure 2D/F) and FlowSOM clustering confirmed these differences (Figure S3B/C, Figure S3E/G). Although different MTs changed between COVID19 and HDs (Figure 2E), only MT7 representing the adaptive NK cell population (CD56^Dim^CD16^+^NKG2A^-^NKG2C^+^CD57^+^) increased in COVID19 compared with HDs and NON-COV-RTI (Figure 2E), confirming the results in conventional flow cytometry analysis. The NK cell IC profiles obtained in the unsupervised mapping (Figure 2F) and in FLowSOM clustering (Figure S3C) were also very different between HDs, NON-COV-RTI and COVID19, identifying ten different NK cell MTs (Figure 2G). Although some differences were found in individual MTs between COVID19, HDs and NON-COV-RTI; only MT5, MT6 and MT7 differentiated COVID19 from both HDs and NON-COV-RTI. MT7 and MT5, reflecting respectively, activated and exhausted NK^CD56Dim^ cells expressing both TIM3 and LAG3 are the predominant MTs in NON-COV-RTI. Both are significantly higher in COVID19 in comparison with HDs and significantly lower in comparison with NON-COV-RTI. MT6, exhausted NK^CD56Bright^ cells expressing all IC, significantly decreased in COVID19 in comparison with HD and increased in comparison with NON-COV-RTI. MT3 (activated NK^CD56Dim^ cells) and MT9 (NKT cells), the most abundant MTs in COVID19, were significantly higher in COVID19 than in NON-COV-RTI. In summary, activated NK^CD56Dim^ cells are different in COVID19 and NON-COV-RTI, characterised by MT3 and MT7, respectively. MT3 presents a higher expression of PD1, TIM3 and CD16 but a lower expression of GzmB and HLA-DR than MT7, which indicates a lower cytotoxic activity.

**Figure 3:**
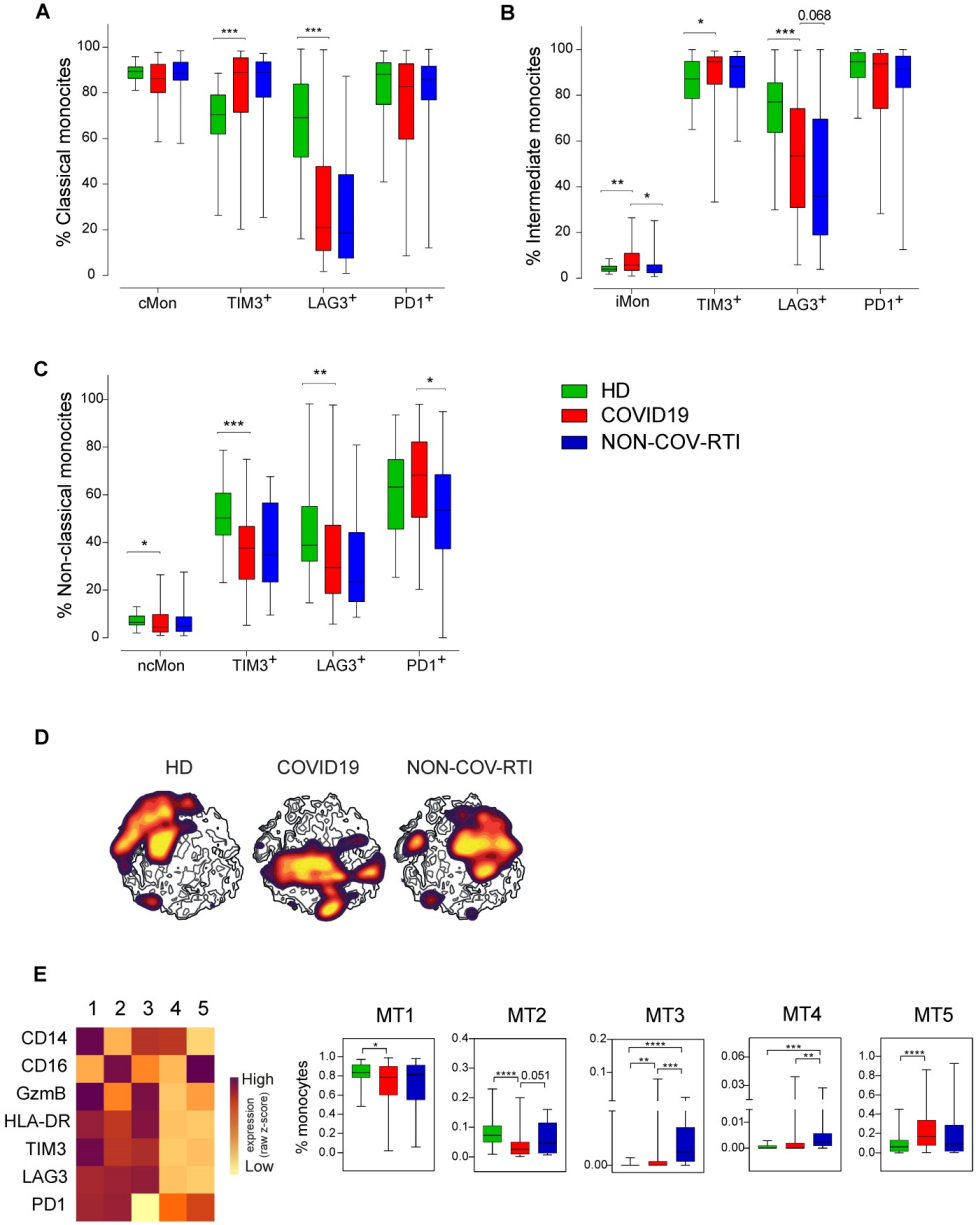
Analysis of the monocyte cell populations in blood from COVID19 patients compared with HD and NON-COV-RTI. The presence of different immune cell populations was analysed in PBMCs by flow cytometry as indicated in methods. **A, B, C)** Expression of cell exhaustion markers (PD1, TIM3 and LAG3) in classical monocyte (A, cMon), intermediate monocyte (B, iMon) and non-classical monocyte (C, ncMon) subsets **D)** viSNE maps of the monocyte populations from COVID19 patients, HD and NON-COV-RTI. **E)** Heat maps showing the MFI in each FlowSOM metacluster (z-scores) of CD14, CD16, GzmB, HLA-DR, TIM3, LAG3 and PD1 in CD14^+^ monocyte cells. The percentage of monocyte cells in each FlowSOM metacluster is shown in the graphs. Boxes represent interquartile ranges (IQRs). Statistical significance was determined by unpaired Mann-Whitney or Kruskal-Wallis tests as indicated in methods: *p < 0.05, **p < 0.01, ***p < 0.001 and ****p < 0.0001.

When the different monocyte subsets were determined (Figure 3A-C) a significant increase in intermediate monocytes (iMon; CD56^-^CD14^+^CD16^+^) of COVID19 patients in comparison with HDs and NON-COV-RTI and a significant decrease in non-classical monocytes (ncMon; CD56^-^CD14^-^CD16^High^) with respect to HDs were found. The frequency of TIM3^+^ cells increased in cMon and iMon while LAG3^+^ cells decreased in COVID19 in comparison with HD. In ncMon, TIM3^+^ and LAG3^+^ cells decreased in COVID19 patients in comparison with HD; similar results were observed in NON-COV-RTI cohort with no differences with COVID19. The frequency of PD1^+^ cells did not change in any monocyte subpopulations with respect to HDs but it decreased in ncMon of NON-COV-RTI (Figure 3C), suggesting that TIM3 and LAG3 expression may be more relevant than PD1 during the regulation of monocyte responses in COVID19. The viSNE representation of the data highlighted key monocyte regions found preferentially in cMon (Figure S3D). The FlowSOM clustering analysis (Figure S3D) identified five different MTs (Figure 3E). MT1 decreased in COVID19 while MT4 increased only in NON-COV-RTI. Both MT1 and MT4 represent cMon although MT1 presents a high expression of GzmB, TIM3, LAG3 and PD1 while MT4 reflect cells expressing only PD1 at a low level. MT3 (iMon: GzmB^High^, HLA-DR^High^, TIM3^High^, LAG3^High^ and PD1^neg^) also increased in COVID19 and NON-COV-RTI patients compared with HDs and this change was also significant in NON-COV-RTI versus COVID19. MT2 frequency decreased in COVID19 while MT5 increased. Both represent ncMon but while MT2 expresses high levels of TIM3, LAG3 and PD1, MT5 only presents intermediate levels of PD1.

### Correlation between immune cell profile and COVID19 severity

**Figure 4:**
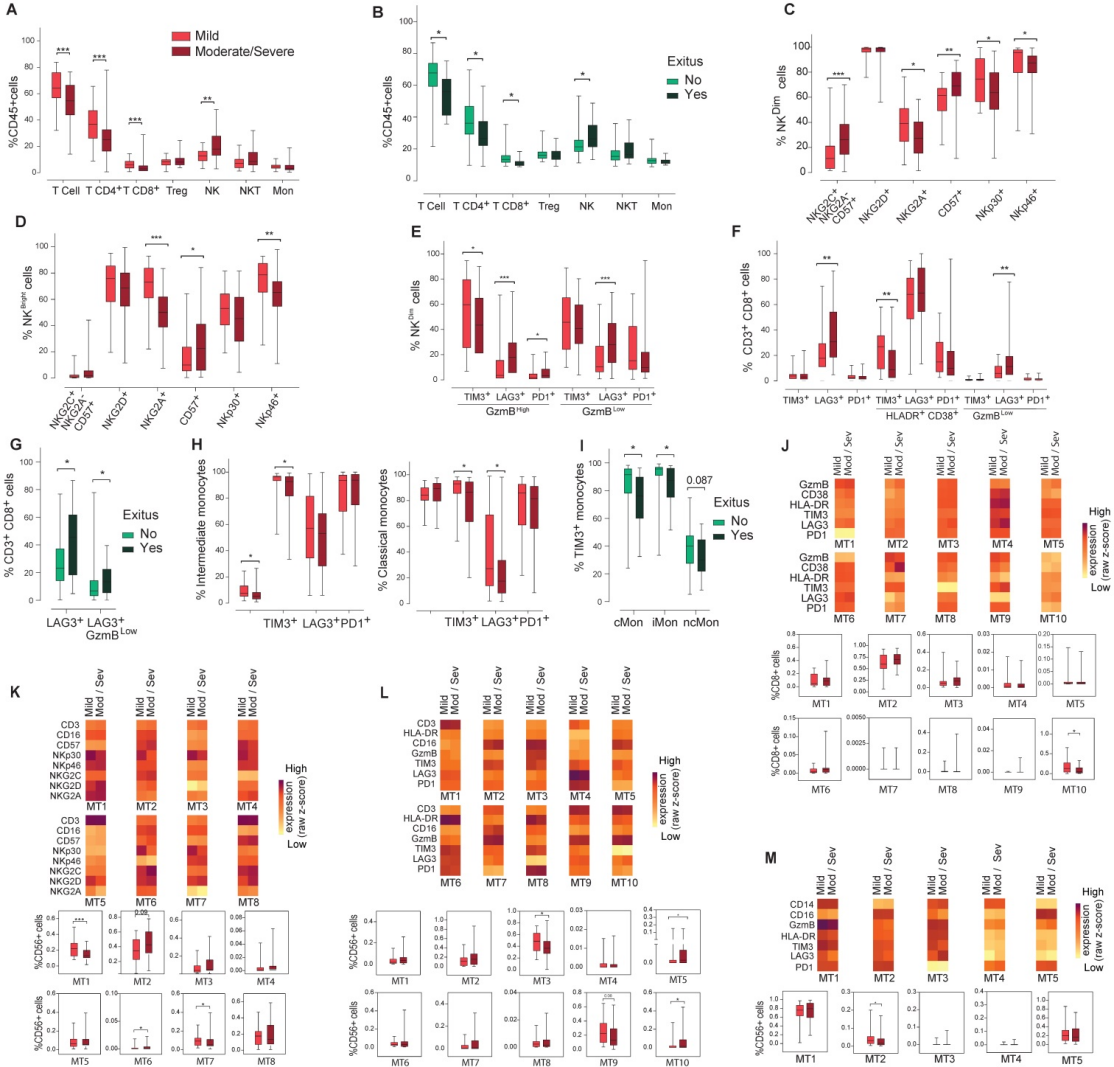
Analysis of the main immune cell subsets in blood from COVID19 patients classified according to severity. The presence of different immune cell populations was analysed in PBMCs by flow cytometry as indicated in methods. **A)** Frequencies of total T, CD4^+^T, CD8^+^T, T^reg^, NKT and NK cells and monocytes in mild and moderate/severe patients. **B)** Frequencies of total T, CD4^+^T, CD8^+^T, T^reg^, NKT and NK cells and monocytes in alive and deceased patients. **C, D, E)** Expression of activation/inhibitory receptors in NK cells in both NK^CD56Dim^ and NK^CD56DBright^ cells and activation/exhaustion markers in the NK^CD56Dim^ cell subset mild and moderate/severe patients. **F)** Expression of activation/exhaustion markers in CD8^+^T cells mild and moderate/severe patients. **G)** LAG3 expression in total and exhausted CD8^+^T cells in alive and deceased patients.** H)** Frequency of iMon and cMon subsets and expression of exhaustion markers. **I)** Expression of TIM3 in monocyte subsets from alive and deceased patients. **J, K, L, M)** Heat maps showing the MFI in each FlowSOM metacluster (z-scores) of activation, inhibitory and exhaustion receptors and markers in CD8^+^T (**J**), CD56^+^ NK (**K, L**) and monocytes (**I**). The percentage of the respective cell populations in each FlowSOM metacluster is shown in the graphs. Boxes represent interquartile ranges (IQRs). Statistical significance was determined by unpaired Mann-Whitney or Kruskal-Wallis tests as indicated in methods: *p < 0.05, **p < 0.01, ***p < 0.001 and ****p < 0.0001.

The lymphopenia observed in moderate/severe cases was reflected in a significant decrease in the frequency of CD3^+^ T cells, both in the CD4^+^ and CD8^+^ T cell subpopulations, while no changes were observed in monocytes or NKT cells (Figure 4A). A significant increase was shown in NK cell percentage in moderate/severe group regarding to the mild group (Figure 4A). The decrease in both subsets of CD3^+^ T cells and the increase in NK cells were also significant in patients who died of COVID19, confirming the differences observed in the severity groups (Figure 4B).

There were no differences between NK^CD56Dim^ and NK^CD56Bright^ cell frequency nor ratio (data not shown). NK cells expressing the inhibitory receptor NKG2A or the activation receptors NKp30 and NKp46 significantly decreased in moderate/severe patients in both NK^CD56Dim^ and NK^CD56Bright^ subpopulations (Figure 4C/D). A significant increase was observed in highly differentiated CD57^+^ NK^CD56Dim^ and NK^CD56Bright^ subpopulations and in the adaptive NK cell population in moderate/severe group (Figure 4C/D), although they were not significantly increased in COVID19 patients who died. In moderate/severe cases there was a significant decrease in TIM3^+^ activated NK cells and an increase in activated PD1^+^ or LAG3^+^ NK cells as well as in exhausted LAG3^+^ NK cells (Figure 4E). It should be remarked that the percentage of LAG3 expressing cells is higher in exhausted NK cells (27.9%) than in activated NK cells (18%), which could indicate a dysfunction in NK cell immune response in moderate/severe COVID19 (Figure 4E).

A significant decrease in the frequency of activated CD8^+^ T cells expressing TIM3 was observed in moderate/severe cases (Figure 4F). However, a marked increase was found in exhausted CD8^+^T cells expressing LAG3, doubling from 6% in mild patients to 12% in moderate/severe patients, and in patients who died (Figure 4G). Interestingly, the frequency of activated CD8^+^ T cells expressing LAG3 did not vary among patients who died or survived (data not shown), which indicates a relative increase in the frequency of exhausted CD8^+^ T cells in comparison with activated ones.

Finally, moderate/severe COVID19 patients presented a significantly lower iMon percentage than mild cases (Figure 4H) which might be related to a lower capacity to present antigens and initiate an immune response against COVID19 (21. Although the rest of monocyte subpopulations did not change (Figure 4H and data not shown), moderate/severe patients present a significant decrease in the percentage of cMon expressing the IC TIM3 and LAG3 and iMon expressing TIM3 (Figure 4H). Strikingly, TIM3 expressing monocytes also significantly decreased in cMon and iMon subsets of patients who died (Figure 4I), suggesting that their decrease could be related with a greater inflammatory potential, increasing disease severity and mortality.

Regarding FlowSOM clustering analysis, in CD8^+^ T cells a statistically significant decrease in the frequency of MT10 was found in moderate/severe vs mild patients (Figure 4J), which represents exhausted cells, supporting the findings by conventional flow cytometry. On the other hand, the analysis of activating and inhibitory receptor patterns on NK (CD56^+^) cells showed a statistically significant decrease in MT1 and MT7 frequency between moderate/severe and mild patients while MT6 frequency increased (Figure 4K). MT2, corresponding to adaptive NK cells (CD16^+^CD57^+^NKG2C^+^NKG2A^Low^) increased in moderate/severe cases although it did not reach statistical significance. Regarding the IC panel (Figure 4L), an increase in the frequency of MT5 and MT10 and a decrease in the frequency of MT3 and MT9 were observed in moderate/severe cases. Within these MTs, the expression of LAG3 increased in moderate/severe cases. In addition, LAG3 was upregulated in many other NK cell MTs (MT2, MT6 and MT7) in moderate/severe patients with respect to mild patients. In the monocyte subsets a significant decrease in MT2 (TIM3^+^PD1^+^LAG3^+^ ncMon) was observed in moderate/severe cases (Figure 4M) confirming the potential role of these IC in controlling monocyte-mediated inflammation during COVID19.

### Immunomodulatory circulating soluble factors in HD, COVID19 and NON-COV-RTI and correlation with COVID19 severity

In contrast to previous studies our analysis concerning soluble immunomodulatory proteins was not only restricted to inflammatory cytokines and chemokines, but in addition, we analysed the presence of T and NK-cell derived serine-proteases GzmA and GzmB, involved in the regulation of inflammatory responses*,* and soluble ligands for the CD8^+^T and NK cell activating receptor NKG2D (ULBP and MIC families) involved in pathogen immune-evasion.

A general increase of inflammatory cytokine and chemokine levels was observed during COVID19 in comparison with HD (Table 1). Although a significant increase was observed in the mean values of IL1 family members, IL1β, IL31, IL33 and IL36B and IFNα in COVID19, the concentration of these cytokines in a lot of patients was low or even undetectable. IFNβ was not detected in COVID19. Only IL18 is detected in most of the patients at relatively high levels, which suggest that it might be the best marker to study the role of IL1 family in COVID19. The level of NKG2D soluble ligands (MICs and ULBPs) and Gzms were significantly increased in COVID19 patients in comparison to HDs (Table 1), a finding that has not been previously reported.

**Figure 5:**
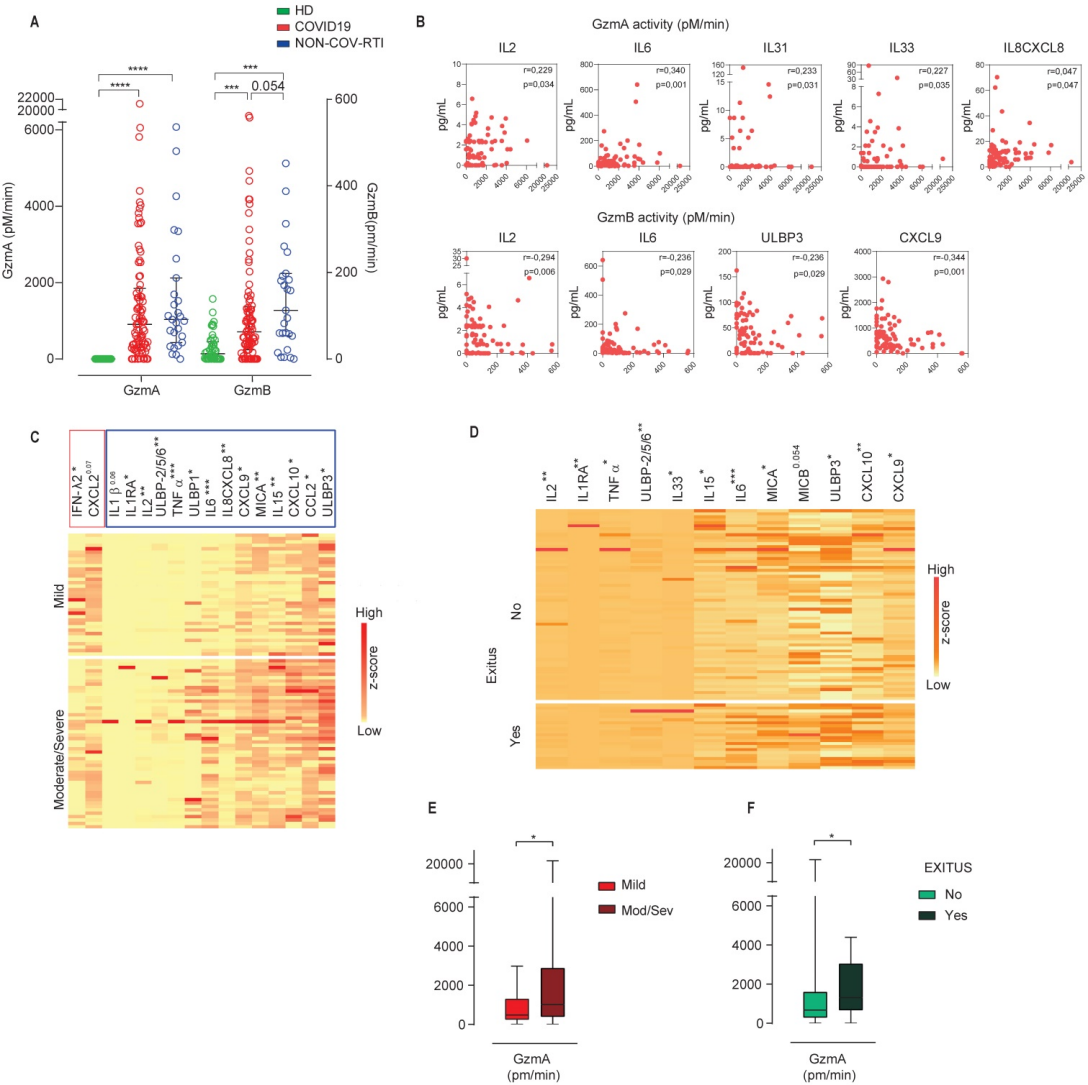
Analysis of soluble GzmA and GzmB in COVID19 patients compared with HD and NON-COV-RTI and correlation with inflammatory cytokines. Gzm concentration and activity were analysed by multiplexed cytokine array and internally quenched fluorescent peptide substrates, respectively, in COVID19, HD and NON-COV-RTI as indicated in methods. **A, B)** GzmA and GzmB activities in serum (**A**) and Spearman correlation between GzmA or GzmB activities and cytokines in serum from COVID19 (**B**). Pearson correlation coefficient (ρ) and p values are indicated. **C, D)** Heat map of the expression of the indicated plasma soluble factors (z scores) from COVID19 patients classified according to either moderate/severe and mild cases (**C**) or alive and deceased patients (**D**). **E, F)** Serum GzmA Activity in COVID19 patients classified according to either moderate/severe and mild cases (**E**) or alive and deceased patients (**F**). Boxes represent interquartile ranges (IQRs). Statistical significance in A and C-F was determined by unpaired Mann-Whitney or Kruskal-Wallis tests as indicated in methods: *p < 0.05, **p < 0.01, ***p < 0.001 and ****p < 0.0001.

As expected, the differences between COVID19 and NON-COV-RTI were less pronounced than in the comparison with HDs, yet several members of the IL1 family such as IL18, IL1RA, IL36B and IL31, IL2, IFNα and TNFα increased significantly in COVID19. In addition, the chemokines involved in T, NK cell and/or monocyte mobilization, CXCL10, CCL2, CCL8 and the soluble NKG2D ligands, MICA and ULBP3 increased significantly in COVID19 compared to NON-COV-RTI (Table 1). There was no difference in other inflammatory factors such as IL6, IL12, IL15, IL7, IL1β, IL10 and CXCL2 between NON-COV-RTI and COVID19 patients. A significant increase in GzmA and GzmB activity (Figure 5A) was found in COVID19 patients only in comparison with HDs (Figure 5A). A significant correlation was observed between GzmA activity, but not concentration, and the inflammatory cytokines IL2, IL6, the IL1 family members, IL33 and IL31, and the chemokine IL8/CXCL8 (Figure 5B). Supporting this correlation, it has been shown that active human GzmA can induce the generation of IL6 and IL8 in several cell types 22, 23. GzmB activity also showed correlation with some soluble factors like the inflammatory cytokines IL2 and IL6, the soluble NKG2D ligand ULBP3 and the chemokine CXCL9 (Figure 5B).

A clear increase was observed in the NKG2D soluble ligands, MICA and ULBPs in moderate/severe cases in comparison with mild cases (Figure 5C) and in the group of patients that died (Figure 5D). The inflammatory cytokine profile also showed significant differences between moderate/severe and mild groups as shown in Figure 5C and 5D. From all of them it should be noted that TNFα, IL2 and IL1RA also showed an increase specifically associated to COVID19 (Table 1). Notably IFNλ2 was significantly downregulated in severe/moderate patients compared to mild ones (Figure 5C), albeit it was not significant in deceased patients (Figure 5D). Within the chemokines that were significantly upregulated in COVID19, CXCL10, CCL2 and CXCL9 together with IL8/CXCL8 increased significantly in moderate/severe cases (Figure 5C) and CXCL10 and CXCL9 in patients who died (Figure 5D), indicating that CXCL10 and CXCL9 differentiate COVID19 from NON-COV-RTI and correlate with severity upon admission and death within COVID19 patients. Finally, moderate/severe patients and those who died presented a significant increase in GzmA activity (Figure 5E/F). Individual violin plots and raw data of the plasma soluble factors is shown in Figures S4 and S5.

### Patient classification and prediction of clinical outcome using multivariate logistic regression analyses

**Figure 6:**
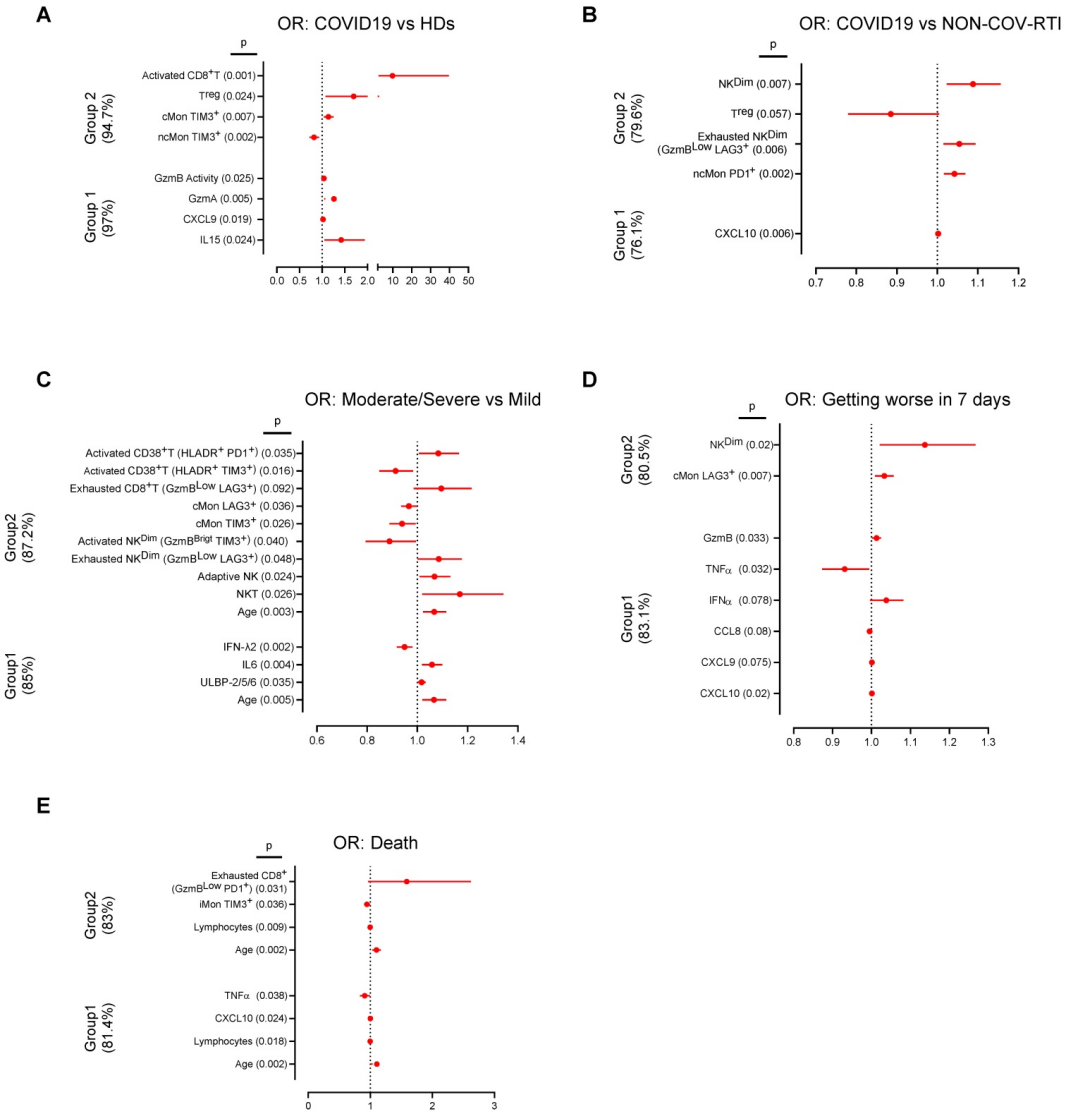
Forest plots depicting the adjusted odds ratios obtained from multivariate logistic regression analysis at hospital admission classified according diagnosis, evolution and severity. Two groups of variables were used, Group 1: Soluble factors, lymphocyte counts and age. Group 2: Cellular immune populations, lymphocyte counts and age. **A, B)** Multivariable logistic regression analysis for the associations of immunological factors and disease groups, COVID19 vs HD (A) and COVID19 vs NON-COV-RTI (B). **C, D, E)** Multivariable logistic regression analysis for the associations of immunological factors and COVID19 severity groups: mild vs moderate/severe (C), worsening (D) and alive or deceased (E). Dotted line indicates the area of the plots where odds ratios are less than 1, indicative of negative associations. Adjusted odds ratios are indicated with points and confidence lines encompass the range between the lower and upper limits. In parent is indicted the percentage of case explained by the logistic regression model.

Finally, we inquired if particular cell populations and immunomodulatory soluble factor expression patterns might help to diagnose and predict clinical outcomes among COVID19 patients (Figure 6). To this aim we used univariate and multivariate logistic regression models in two groups of data sets as indicated in the methods section of Supplementary Information. The results of the analyses, including odds ratios and confidence intervals, for COVID19, HD and NON-COV-RTI classification are shown in Table S6 and Figure S6A/B. Within Group 1, four variables (IL15, CXCL9, GzmA and GzmB activity) accurately classified 97% of COVID19 diagnosis compared with HDs. In Group 2, also four variables (TIM3^+^cMon and TIM3^+^ncMon, T^reg^ cells and activated CD8^+^ T cells) classified 94.7% of patients. One variable in Group 1 (CXCL10) and four variables (PD1^+^ncMon, exhausted LAG3^+^NK cells, T^reg^ and NK^CD56Dim^ cells) in Group 2 classified 76.1% and 79.6% of the patients between COVID19 and NON-COV-RTI, respectively.

The results concerning disease severity, worsening and death are shown in Table S7 and Figure S6C-E. In Group 1, four variables (age, ULBP-2/5/6, IL6 and IFNλ2) were found to classify 85% of moderate/severe COVID19. In group 2, eleven variables, including age, activated and exhausted CD8^+^T cells and adaptive NK cells were found to classify 87.2% of moderate/severe COVID19. In group 1, six variables (CXCL10, CXCL9, CCL8, IFNα, TNFα and GzmB) classified 83.1% of patients that got worse after 7 days. In Group 2, only 2 variables, NK^CD56Dim^ and LAG3^+^cMon, classified 80.5% of patients that got worse. Finally, in the prediction models of patient death, four variables in group 1 (Age, lymphocyte counts, CXCL10 and TNFα) predicted 83.0% of the patients that died. The same prediction value was obtained with four variables in group 2, (age, lymphocyte counts, exhausted PD1^+^CD8^+^T cells and TIM3^+^iMon). The Area Under the Curve Tables S6 and S7) showed good (0.7-0.9) or very good (>0.9) significant values and the p values obtained in the Hosmer and Lemeshow test were always higher than 0.05 confirming the utility and the fit goodness of the different models.

## Discussion

In this study, we present comprehensive immune profiles for human SARS-CoV-2 infection causing COVID19. By employing a diverse antibody panel and the Flowsom clustering techniques to systematically discover sub-populations and their frequencies in these data, we found a heterogeneous immune response in COVID19 characterised by the concomitant presence of activated and exhausted CD8^+^ T and NK cells. The presence of these cell populations is concomitant to an increased level of soluble inflammatory cytokines and chemokines as well as soluble ligands involved in the regulation of the antiviral activity of NK/CD8^+^T cells that partly explain an inefficient antiviral CD8^+^T/NK cell activity despite the presence of highly activated cells in severe patients that finally died. Among the different factors that might account for this inefficient deregulated immune response, age and ACE2 expression might be critical ones as previously described (24, 25. Indeed, although we did not determine ACE2 expression we also found that increased age as well as different comorbidities correlated with disease severity.

Our results provide robust and detailed information on the effects of COVID19 infection on immune response and a number of novel findings, including host immunity-based models with potential utility for diagnosis and prognosis in COVID19. Notably, we have identified specific immune signatures in blood characteristic of COVID19 patients, which are not only different from HD but in addition, they differ from other respiratory infections (NON-COV-RTI cohort), a question that remains poorly explored in COVID19.

Our findings indicate a prominent CD8^+^T, NK cell and monocyte activation during the acute immune response in COVID19. According to our data there is a strong activation of CD8^+^T and NK cells with a high presence of adaptive and terminally differentiated NK cells and increased expression of NKG2D, correlating with disease severity. An excessive NK cell activation in COVID19 could amplify the systemic inflammatory response resulting in increased physiological dysfunction and organ injury as described for sepsis 26-28. Our results confirms and expand previous studies 29 by providing a number of new findings that contribute to explain the limitation of activated NK/CD8^+^T cells to control SARS-CoV-2 infection, a concept that is not clear yet 16, 17, 20, 30, 31. NK and CD8^+^T cell activation seems to be counterbalanced by the greater increase in exhausted cells observed in moderate/severe COVID19, with LAG3 IC being the most relevant one. This result agrees with previous findings showing an exhausted CD8^+^T cell phenotype in COVID19 patients, albeit those studies were mainly focused on PD1 expression and COVID19 was only compared with HDs 17, 32, 33. In contrast, our findings provide new insights into the phenotype of bona fide (GzmB^Low^) exhausted NK and CD8^+^T cells showing a more complex scenario where different IC, including soluble immunomodulatory ligands like MIC and ULBP family members, contribute to cell exhaustion and inactivation. Strikingly, when the flow cytometry data were considered by unsupervised analysis, we found different cell clusters resembling exhausted cells with a heterogeneous expression of TIM3, PD1 and LAG3 characterised by single cells expressing more than one IC, creating cellular fingerprints that differ between COVID19 and other respiratory infections (NON-COV-RTI). In addition, and in agreement with a recent study focused on PD1 34, 35, our results reveal that a large proportion of CD8^+^T cells expressing IC are not functionally exhausted but they are activated cells that express activation (HLA-DR, CD38) and cytotoxic (GzmB) markers. Our results expand this concept to NK cells indicating that a large proportion of NK cells expressing IC are not exhausted but activated cells. Yet these activated cells seem not to be able to control SARS-CoV-2 infection, which could be due to the presence of high levels of soluble CD8^+^T/NK cell ligands with immunosuppressive activity like MIC and ULBP family members, detected in moderate/severe COVID19 and deceased patients. Soluble NKG2D ligands have been shown to modulate NK cell responses, inhibiting NK cell activity in cancer and infection 36.

Our results present potential implications for the use of immune checkpoint inhibitors (ICIs) for COVID19 treatment, suggesting that LAG3 blockade could be more effective that PD1 blockade to increase immune response against SARS-CoV-2. However, our data showing the expression of several IC in single cells also suggest that targeting IC to enhance antiviral NK/CD8^+^T cell responses might be challenging as blocking several IC simultaneously might be required. Although functional assays employing IC inhibitors are necessary to confirm this hypothesis, this concept is supported by the results in cancer immunotherapy showing increased efficacy when several IC are simultaneously targeted 37. As an additional level of complexity, the increased amounts of soluble NKG2D ligands, MICs and ULBPs, in blood from moderate/severe COVID19, suggest that blocking LAG3, TIM3 and/or PD1 still might not be sufficient to generate optimal antiviral responses during COVID19. In contrast to CD8^+^T and NK cells, a decrease in the frequency of different monocyte subsets expressing IC is observed in moderate/severe COVID19 patients, which might enhance their proinflammatory capacity contributing to COVID19 pathogenesis as shown in sepsis 38-40. Thus, altogether our data suggest that the potential beneficial effects of targeting IC in COVID19 might be counterbalanced by activation of detrimental monocyte-mediated inflammation, albeit further experimental evidences will be required to confirm this hypothesis.

Our data fit with an overaggressive immune response and the upregulation of inflammatory cytokines in a subset of patients who developed a more severe disease. Indeed, similar to other studies we also found elevated serum cytokines in plasma from moderate/severe COVID19 11, 12, 15, 29. Expanding all these findings and providing new insights into the role of soluble immunomodulators during COVID19, we show that soluble ligands of the CD8^+^ T and NK cell activating receptor NKG2D from the MIC and ULBP families are specifically increased in moderate/severe COVID19 and in patients that die confirming the importance of NK cell regulation during COVID19. The soluble markers analysed define an immunological profile in COVID19 patients that is different from that in other infections and, in addition, differ between mild and moderate/severe cases. Notably, among all factors analysed it is striking that the chemokines CXCL10 and CXCL9 increase in COVID19 in comparison with NON-COV-RTI and, in addition, they are increased in moderate/severe COVID19 and patients who end up dying. Thus, targeting the chemokine axes CXCL9, CXCL10, which are involved in T and NK cell mobilisation, might be helpful to treat COVID19 as previously found in experimental sepsis 41. Other inflammatory molecules such as IL6, IL12 or IL15 do not appear to be specific of COVID19, although their presence could correlate with COVID19 severity. In addition, our findings reveal a previously unrecognised potential role of Gzms, especially GzmA, in the pathology of COVID19, more specifically in upregulation of the inflammatory cytokine response. This result is supported by recent findings showing that GzmA is involved in the pathogenesis of bacterial and polymicrobial sepsis 23, 42. Intriguingly, GzmA or GzmB activity did not correlate with their serum concentration, suggesting that inactive forms of Gzms are present in circulation. This is not surprising and has been previously reported 43. Indeed, Gzms activity is regulated by extracellular inhibitors present in blood, some of which have been found to be downregulated during inflammatory responses like sepsis. This might explain the lack of correlation between Gzm concentration and activity during inflammation, and, as expected, that the active proinflammatory forms of Gzms, but not the total amount of Gzms, correlate with disease severity and the upregulation of inflammatory cytokines. Further experiments including animal models will be required to confirm the potential role of active GzmA and/or GzmB in COVID19.

In addition to provide a major understanding on the immune response associated with COVID19 pathogenesis, our study has allowed us to generate models based on cell immunity and soluble immunomodulators to differentiate COVID19 from other respiratory infections, classifying them according to severity and predicting the risk of death, importantly early at the moment of hospital admission. Strikingly, among other soluble factors, CXCL10 is present in all models classifying patients in COVID19 or NON-COV-RTI, moderate/severe COVID19 and death risk. In addition to modulate T and NK cell migration, CXCL10 has been related to thrombosis and correlated with the coagulation parameters in COVID19, although it did not show differences between severe/critical cases who either survive or die 44, 45. Regarding immune cells, the adaptive NK cell subset and different populations of exhausted cells appear in the different models confirming their importance to explain COVID19 severity in some patients. Some of these models can be very useful to help in clinical decisions, stratifying patients according to prognosis and anticipating treatments for those in whom a worse evolution is expected.

The prospective character of our study using a large number of patients (87) strengthens the validity of the conclusions and of the models generated. However, these models will require validation in larger independent cohorts of COVID19, which is one of the main limitations of this study. In addition, the differences of age in HDs (44±12.5) and COVID19 (71.2±17.9) groups could introduce a bias in data analyses as age might influence the presence of different immune cell subsets. However, we have repeated all cell analyses using an independent old HD cohort (mean age 71.2) and have found similar results discarding this potential problem. In addition, all samples from patients included in this study were collected during the first 24h after Hospital entrance, before any immunosuppressive treatment was prescribed, discarding any influence of these type of treatments in our analyses.

Another limitation is the lack of specific diagnosis in the NON-COV-RTI group. Unfortunately, during the period the samples were taken, in the midst of the first COVID19 wave that strongly hit our country, specific diagnosis for other respiratory infections were not performed and respiratory samples were not stored at the Hospital due to logistic reasons. Last but not least, it would have been very useful to identify the specific infections in the NON-COV-RTI cohort, albeit these are infections that are not commonly identified in hospitalised patients. Thus, we could say that our classification represents a real-life situation in which COVID19 confirmed cases requires differentiation from other infections with similar symptoms for therapeutic but also logistic reasons.

## Supplementary Material

Supplementary materials and methods, figures and tables.

## Figures and Tables

**Table 1:** Comparison of the cytokine and chemokine levels (median and interquartile ranges) of COVID19 with HD and NON-COV-RTI. Statistical significance was determined by unpaired Mann-Whitney: *p < 0.05, **p < 0.01, ***p < 0.001 and ****p < 0.0001.

	P (HDs vs COVID19)	HDs			COVID19			NON-COV-RTI			P (COVID19 vs NON-COV-RTI)
		median	Q1	Q3	median	Q1	Q3	median	Q1	Q3	
MICA	0,002	28,5	14,0	70,3	57,9	31,1	150,1	29,6	6,5	47,4	0,006
MICB	0,000	29,3	24,2	42,5	78,1	40,4	109,7	55,2	40,1	81,4	ns
ULBP1	ns	0,0	0,0	0,0	0,0	0,0	0,0	0,0	0,0	0,0	ns
ULBP-2/5/6	0,099	0,0	0,0	23,6	0,0	0,0	71,7	0,0	0,0	54,5	ns
ULBP3	0,002	3,4	0,0	25,4	31,6	0,0	68,6	8,9	0,0	34,5	0,035
IL12	0,003	0,0	0,0	0,0	0,0	0,0	55,2	0,0	0,0	39,2	ns
IL25	ns	0,0	0,0	61,6	0,0	0,0	44,6	0,0	0,0	32,8	ns
IL2	0,002	0,0	0,0	1,1	1,0	0,0	2,4	0,0	0,0	2,3	0,024
IL15	0,000	0,8	0,0	1,2	3,4	1,6	7,7	2,4	0,5	4,7	ns
IL6	0,000	0,7	0,4	1,5	15,3	5,6	51,8	7,4	2,5	33,3	ns
IL1 B	0,002	0,0	0,0	0,1	0,1	0,0	1,3	0,3	0,0	1,4	ns
IL18	0,000	219,5	180,8	305,3	533,7	339,1	808,4	312,2	225,6	673,3	0,006
IL1RA	0,000	360	250	660	3400	1800	8400	1600	790	5000	0,017
IL36 B	0,000	1,0	0,3	1,5	3,7	1,0	5,7	0,9	0,2	1,9	0,000
IL33	0,003	0,0	0,0	0,0	0,0	0,0	0,8	0,0	0,0	0,6	ns
IL31	0,002	0,0	0,0	0,0	0,0	0,0	0,1	0,0	0,0	0,0	0,042
IL7	0,048	2,3	1,2	3,2	2,9	1,5	5,3	2,6	1,4	4,5	ns
IL10	0,001	0,0	0,0	0,8	1,3	0,0	6,0	0,0	0,0	2,6	ns
IL8 CXCL8	0,000	2,7	1,3	4,0	7,4	3,7	12,4	4,7	3,7	16,1	ns
CXCL10	0,000	33,0	20,7	54,2	346,1	61,2	1181,4	61,5	50,4	165,8	0,002
CCL2	0,000	122,3	97,0	158,7	243,6	150,1	379,7	142,6	92,8	305,6	0,034
CXCL2	0,016	185,5	68,0	337,4	281,2	114,5	714,5	389,1	106,3	712,8	ns
CXCL9	0,000	0,0	0,0	210,7	597,2	305,0	846,9	388,8	110,9	671,2	0,059
CCL8	0,000	11,3	2,4	26,0	81,2	36,7	164,5	24,9	16,3	54,0	0,000
IFN-λ3	ns	23,5	0,0	45,5	17,3	0,0	48,9	1,5	0,0	31,0	ns
IFN-λ2	ns	0,0	0,0	24,2	0,0	0,0	43,0	0,0	0,0	43,0	ns
IFNα	0,000	0,0	0,0	0,0	1,7	0,0	9,2	0,0	0,0	0,0	0,000
IFNγ	0,000	0,0	0,0	0,0	1,8	0,0	10,6	0,0	0,0	12,5	ns
IFNβ	ns	0,0	0,0	0,0	0,0	0,0	0,0	0,0	0,0	0,0	ns
FAS L	ns	16,1	12,2	21,2	18,3	11,4	26,1	20,7	15,4	32,6	ns
TNFα	0,000	2,0	1,3	2,7	5,9	4,2	9,7	3,6	3,3	6,7	0,007
TRAIL	0,000	77,3	50,7	99,1	36,4	15,8	70,5	23,1	5,9	40,0	0,025
GRANZYME A	0,000	12,8	2,1	20,2	42,1	24,8	50,9	33,6	14,7	47,4	ns
GRANZYME B	0,000	0,0	0,0	3,7	14,0	7,4	21,2	9,4	1,4	12,7	0,011

## Abbreviations

metaclusters): NON-COV-RTI (non-COVID19 respiratory tract infections
