# Supplementary material for: Integrated analysis of circulating immune cellular and soluble mediators reveals specific COVID19 signatures at hospital admission with utility for prediction of clinical outcomes
Source: Theranostics. 2022 Jan 1;12(1):290–306. doi: 10.7150/thno.63463 (PMC8690910; doi:10.7150/thno.63463)
Supplement: Supplementary file 1 — Supplementary materials and methods, figures and tables. [file thnov12p0290s1.pdf]

SUPPLEMENTARY INFORMATION

Contains

- ***Supplementary materials and methods***
- ***Supplementary figure legends***
- ***Supplementary table legends***

Supplementary materials and methods

Sample processing

Peripheral blood was collected into sodium heparin tubes and centrifuged for 10 min at 2500 rpm at room temperature (RT) to separate the cellular fraction from the plasma. The plasma was removed from the cell pellet and stored at -80 ºC for posterior use. The cell pellet was diluted 1:1 in RPMI 1640 medium and carefully added to Histopaque-1077 (Sigma) for peripheral blood mononuclear cell (PBMC) isolation by centrifugation at 2500 rpm at RT for 10 min. PBMC layer was collected, washed with RPMI 1640, counted and aliquoted for staining and flow cytometry analysis. All flow cytometry analyses were performed using fresh PBMCs.

Flow cytometry

For the surface staining, PMBCs were resuspended in 50 µl PBS with 5 % FCS and stained with the different antibody cocktails for 20 min at 4 ºC in dark, washed twice with PBS + 5 % FCS and then fixed for 30 min at 4 ºC in the dark using 2% PFA. Following surface staining, cells that required intracellular staining were fixed/permeabilized for 30 min at 4 ºC in the dark using the FoxP3 transcription factor buffer kit (Miltenyi). Following fixation/permeabilization, cells were washed twice with permeabilization buffer, resuspended in 50 µl permeabilization buffer and stained with intracellular antibodies for 30 min at 4 ºC in the dark. Samples were washed twice with permeabilization buffer following staining and fixed. All samples were acquired on a Gallios (Beckman Coulter) Flow Cytometer. The list of the antibodies used for immune cell phenotyping was: Miltenyi Biotec, CD14-VioBlue (130-110-524), CD16-FITC (130-113-392), CD25-APC (130-113-280), CD3-VioGreen (130-113-134), CD38-FITC (130-113-426), Treg detection kit CD4/CD25/CD127 (130-096-082), CD56-PerCP Vio700 (130-100-681), CD57-APC-Vio770 (130-111-813), CD8-PerCP Vio700 (130-110-682), FoxP3 Staining Buffer Set(130-093-142), GzmB-PE (130-116-486), HLADR-APC-VIO770 (130-111-792), LAG3-APC (130-105-453), NKG2A-PE-VIO615 (130-120-035), NKG2C-PE (130-103-635), NKP46-APC (130-092-609), TIM3-PE vio770 (130-121-334); Biolegend,NKp30-PE/Cy7 (325214), PD1-Alexa Fluor700 (329952), CD45-Brilliant Violet 421 (304032); BD,NKG2D-BV421 (743558).

High dimensional flow cytometry data analysis

viSNE and FlowSOM (Self-organizing map) analyses were performed using Cytobank (https://cytobank.org). We used t-distributed stochastic neighbouring embedding (t-SNE) to reduce the dimensionality of the cell marker datasets generated using the antibody panels indicated above. FlowSOM clustering analysis compared expression of cell markers was used to identify each cluster and perform an unbiased analysis of the PBMC immunophenotyping data. $CD56^+$ cells, $CD56^+$ or $CD14^+$ cells and $CD3^+CD8^+$ cells from FACS panels 2, 3 and 4 respectively, were analysed separately. SOM was generated using equal sampling of at least 1000 cells from each FCS file and hierarchical consensus clustering by the following markers: CD3, CD16, CD57, NKp30, NKp46, NKG2C, NKG2D and NKG2A for panel 2 analysis; CD14, CD3, HLA-DR, CD16, GZMB, TIM3, LAG3, PD1 or CD56,CD3, HLA-DR, CD16, GZMB, TIM3, LAG3, PD1 for panel 3 analysis and GzmB, CD38, HLA-DR, TIM3, LAG3, PD1 for panel 3 analysis. For each SOM, 100 clusters and 5, 8 or 10 metaclusters (MTs) were identified for panel 2, panel 3 and 4, which were represented in Minimum Spanning Trees (MTS).

Multiplex plasma protein analyses

Luminex assay was run according to manufacturer's instructions in 100 µl of plasma, using a custom human cytokine panel (R&D Systems, catalogue no. LXSAHM). The next proteins were included: IFNα, IFNβ, IFNγ, IL28A/IFNλ2, IL28B/IFNλ3, IL2, IL1β,IL18/IL1F4,IL1RA,IL33, IL36b/IL1F8, IL7, IL10, IL31, IL6, IL12/IL23 p40, IL15, IL17E/IL25, IL8/CXCL8, CXCL10/IP10, CCL2/MCP1, CCL8/MCP2, CXCL9/MIG, CXCL2/MIP2α, MICA, MICB, ULBP-1, ULBP-2/5/6, ULBP-3, TNFα, GzmA and GzmB. Supernatants were mixed with beads coated with capture antibodies and incubated on a 96 well filter plate for 2 hours. Beads were washed and incubated with biotin-labelled detection antibodies for 1 hour, followed by a final incubation with streptavidin-PE. Assay plates were measured using a Luminex 200 instrument (ThermoFisher, catalogue no. APX10031). Data acquisition and analysis were performed using xPONENT software. The standard curve for each analyte had a five-parameter R2 value > 0.95 with or without minor fitting using xPONENT software.

Granzyme activity assay in serum

Serum samples were used to evaluate the activity of both GzmA and GzmB using specific quenching FRET fluorescent substrates (FAM-VANRSAS-DABCYL and FAM-IEPDNLV-DABCYL peptides, respectively). In a nutshell, 40 µl of 100 mM Tris-HCl pH 8.5 or 100 mM Tris-HCl 50 mM NaCl pH 7.8 (buffers for GzmA or GzmB respectively) were added to flat bottom, black plates, with 10 µl of the serum samples. 50 µl of GzmA or GzmB substrates were added and

the fluorescence of the plate was read at time zero and 1 h for GzmA and 24 h for GzmB using 475 nm excitation and 520 nm emission wavelenghts. Gzm activity was calculated based on a calibration curve with known concentrations of carboxyfluorescein.

Statistics

To minimize inter-experimental variability and batch effects between patients, all PBMC samples were acquired, processed, and freshly analysed during four consecutive weeks from April to June 2020. Serum and plasma samples were frozen at -80ºC and later on all of them were thawed and analysed at the same time. Univariate and multivariate logistic regression models were developed using two different groups of variables, representing soluble and immunomodulatory factors (Group 1) or cell populations (Group 2) shown in Table S5. Age, sex and lymphocyte counts were included in all groups except for the comparison between HD and COVID19, since these variables were not known in HDs. First, a univariate logistic regression analysis was performed in the corresponding groups. Variables included in the multivariate discriminant analysis were those with a value of $p < 0.1$ in the univariate logistic regression analysis and / or with a value of $p < 0.1$ in the medians comparison tests. The univariate statistic test used has been chi-square or Fisher exact test for qualitative variant comparison and Mann-Whitney (comparison of two groups of variables) or Kruskal-Wallis (comparison of more than two groups of variables) for quantitative variant comparison. The post-test used was Benjamini, Krieger and Yekutieli test. Variable normality has been analysed with Kolmogorov-Smirnov test and Rho's Spearman has been calculated as correlation coefficients. Statistical models were developed to predict COVID19 of diagnostic and severity. A multivariate logistic regression and discriminant analyses were performed to develop predictive models. Area Under the Curve (AUC), OR and CI95% values were reported for significant variables. Nagelkerke R2 was calculated to analyse sample variability and Hosmer-Lemeshow test was performed to analyse goodness of fit for the logistic regression model. Hosmer-Lemeshow p values higher than 0.05 indicate an adequate calibration of the predictive model. The statistics software used was GraphPad Software 7.0, (Inc. San Diego, CA) and SPSS 26.0 (IBM Corp., Armonk, NY).

Supplementary figure legends

Supplementary Figure 1. Flow Diagram of the progress through the phases of the study. Patient enrolment differed between HDs who followed inclusion criteria, and patients separated into NON-COV-RTI and COVID19 depending on the qRT-PCR or serological test results. Samples were taken and processed and the data analysis divided into univariate statistics test and multivariate logistic regression.

Supplementary Figure 2. Flow cytometry gating strategy. A time gate was initially applied to exclude any electronic noise followed by a singlet gate to exclude doublets. Subsequently total lymphocytes were first gated on a forward scatter (FS)/side scatter (SS) plot and then gated on $CD4^+$ and $CD8^+$ T cells ($CD3^+CD4^+$, $CD3^+CD8^+$), Treg cells ($CD3^+CD4^+CD25^+CD127^-$), NK cells ($CD3^-CD56^+CD16^{+/-}$) and monocyte subsets ($CD3^-CD56^-HLA\text{-}DR^+CD14^{+/-}CD16^{+/-}$). Data were analysed using Kaluza software.

Supplementary Figure 3. Minimum Spanning Tree (MST) of the clusters generated by FlowSOM algorithm of A) $CD8^+$T cell, B, C) NK cells and D) Monocytes. Node diameter is proportional to the number of events. The background colours group nodes into cell types corresponding to different major immune cell types (FlowSOM Metaclusters).

Supplementary Figure 4. Expression of the plasma soluble factors from COVID19 patients classified according to either moderate/severe or mild cases. Individual violin plots (**A**) and raw data (**B**) leading to heat map of Figure 5C. Dashed lines represent median and dotted lines represent interquartile ranges (IQRs). Statistical significance was determined by unpaired Mann-Whitney or Kruskal-Wallis tests as indicated in methods: $*p < 0.05$, $**p < 0.01$ and $***p < 0.001$.

Supplementary Figure 5. Expression of the plasma soluble factors from COVID19 patients according to their exitus status. Individual violin plots (**A**) and raw data (**B**) leading to heat map of Figure 5D. Dashed lines represent median and dotted lines represent interquartile ranges (IQRs). Statistical significance was determined by unpaired Mann-Whitney or Kruskal-Wallis tests as indicated in methods: $*p < 0.05$ and $**p < 0.01$.

Supplementary table legends

Supplementary Table 1. Overview of the patient cohort comprised in the study that includes COVID19 patients, healthy donors (HD) and patients with flu-like symptoms (NON-COV-RTI). Demographic parameters such as age, gender and race are included for each group considered, besides hospitalization characteristics for patients.

Supplementary Table 2. Hospitalization classification for COVID19 clinical severity in three different groups; mild, moderate and severe depending on hospitalization parameters. Demographic parameters such as gender, race and age; and overview of hospitalization characteristics including lymphocyte count, days since symptoms onset and duration of hospital stay in the COVID19 patient groups split in mild and moderate/severe in this study.

Supplementary Table 3. Comorbidity and immunosuppressive treatments information of COVID19 patients comparing mild and moderate/severe classification.

Supplementary Table 4. Comorbidity and immunosuppressive treatments information of COVID19 patients in comparison with NON-COV-RTI patients.

Supplementary Table 5. Variables included in Group 1 and 2 to the Multivariable logistic regression analysis.

Supplementary Table 6. Multivariable logistic regression analysis for the associations of immunological factors and disease groups (COVID19 vs HD and COVID19 vs NON-COV-RTI). Group 1: Soluble factors, lymphocyte counts and age. Group 2: Cellular immune populations, lymphocyte counts and age. The number under each group is the perdition of the model. AUC: area under the curve.

Supplementary Table 7. Multivariable logistic regression analysis for the associations of immunological factors and severity groups of COVID19 patients (mild vs moderate/severe, patients who got worse and alive or deceased). Group 1: Soluble factors. Group 2: Cellular immune populations. The number under each group is the prediction of the model. AUC: area under the curve.

SUPPLEMENTARY FIGURE 1

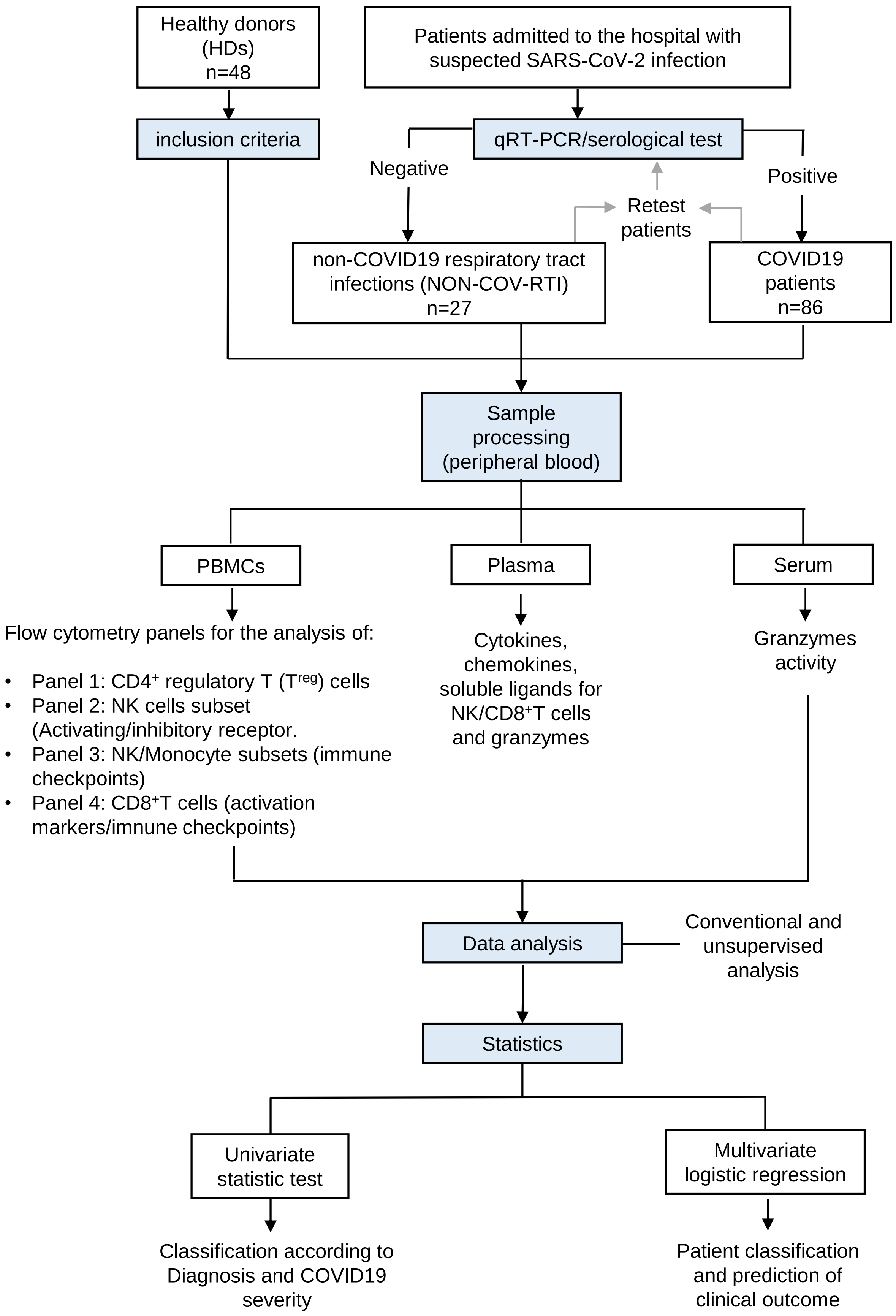

SUPPLEMENTARY FIGURE 2

A)

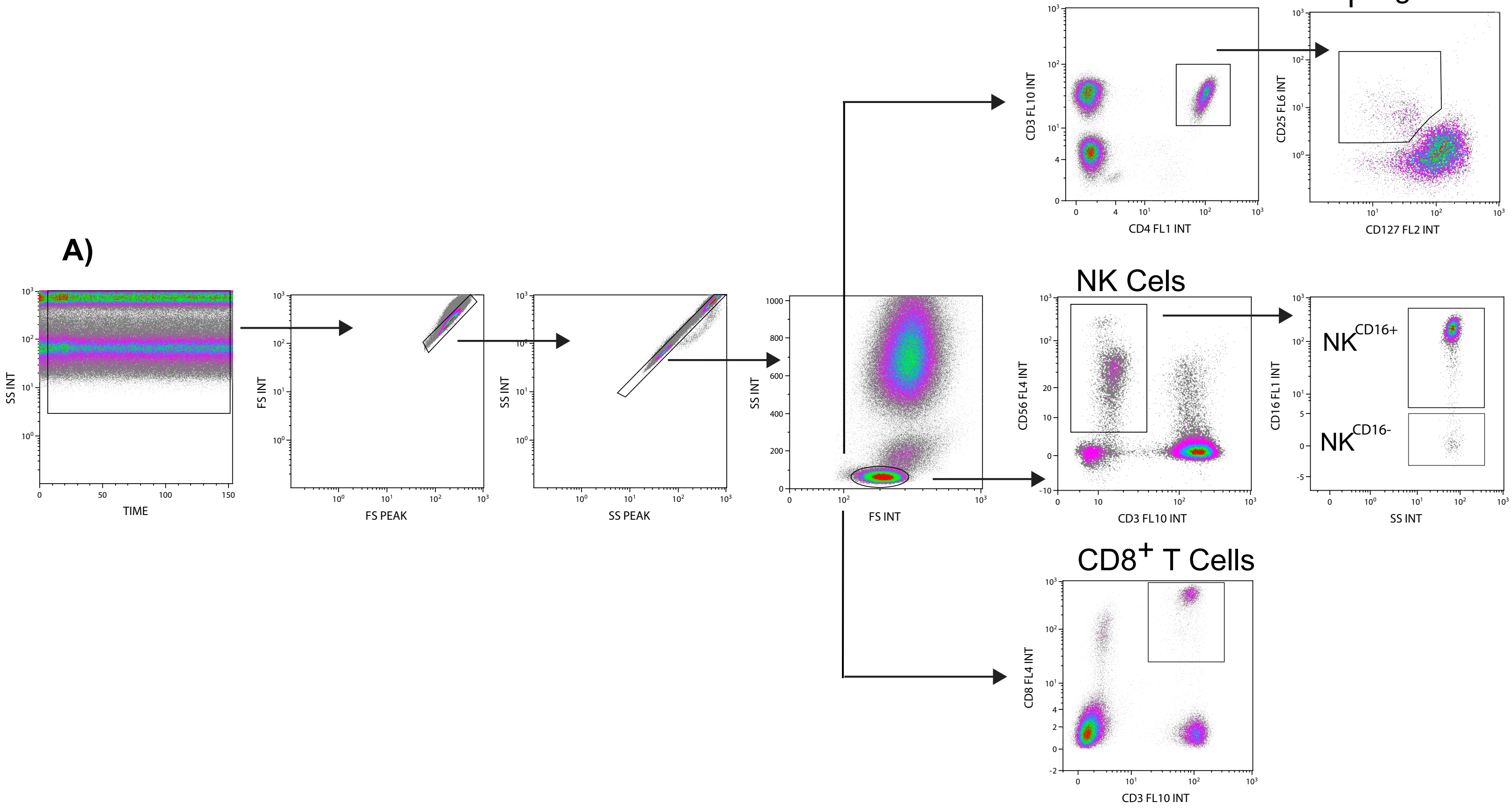

B)

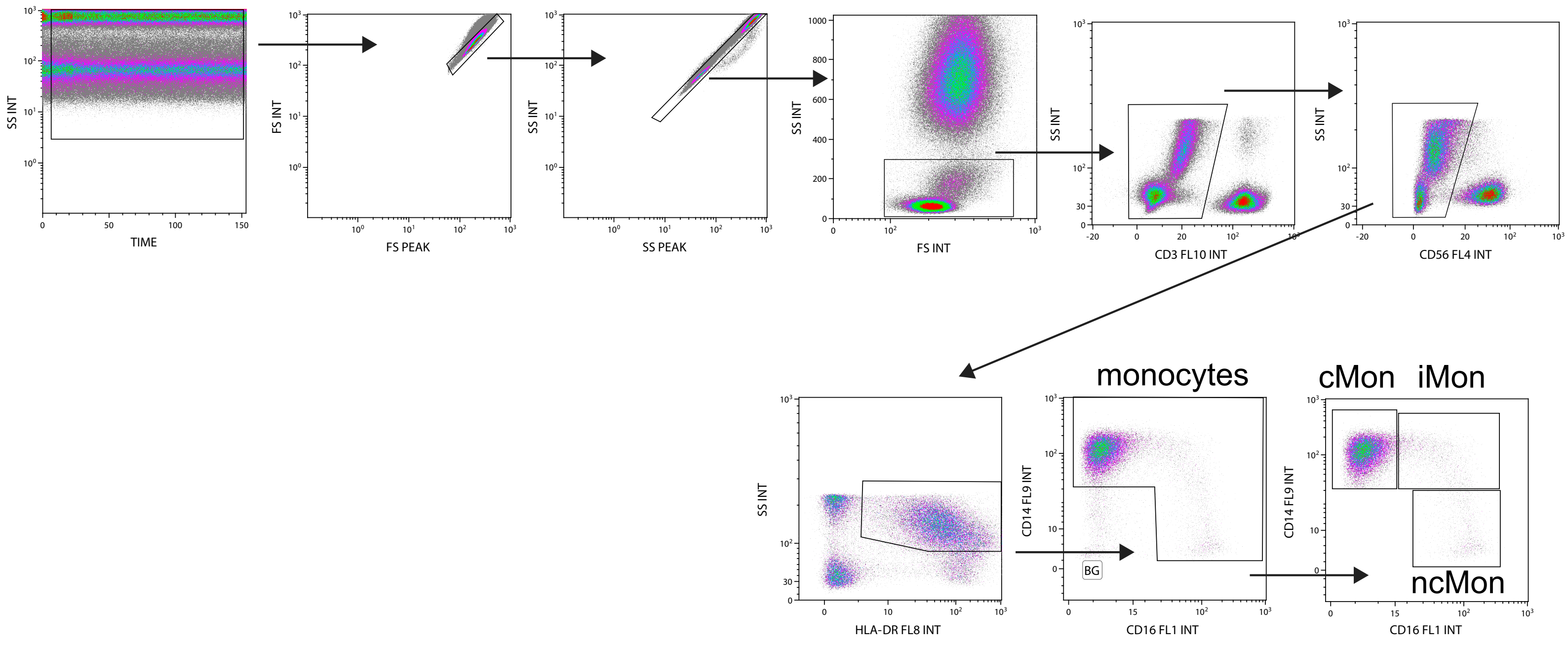

SUPPLEMENTARY FIGURE 3

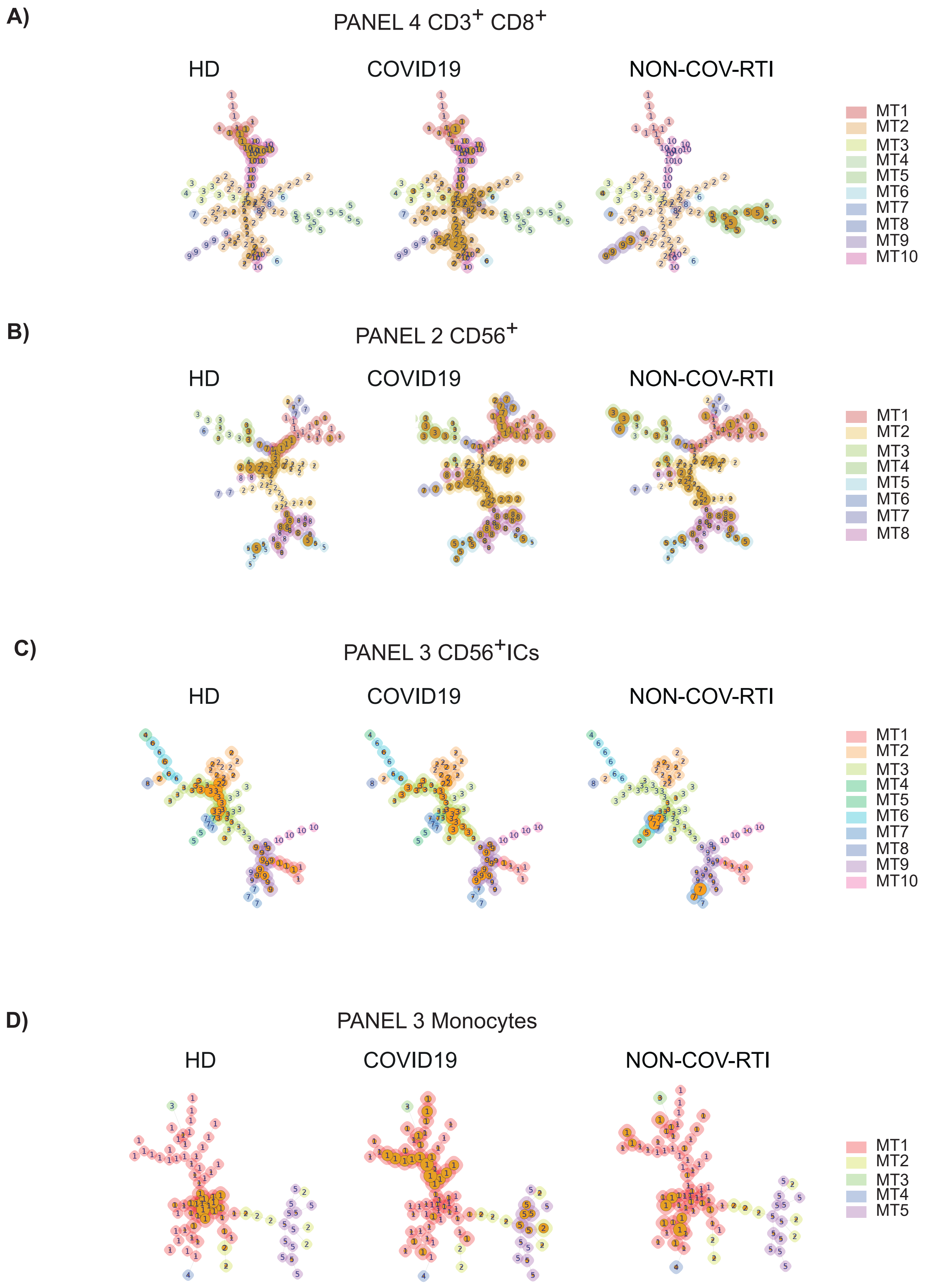

SUPPLEMENTARY FIGURE 4

A)

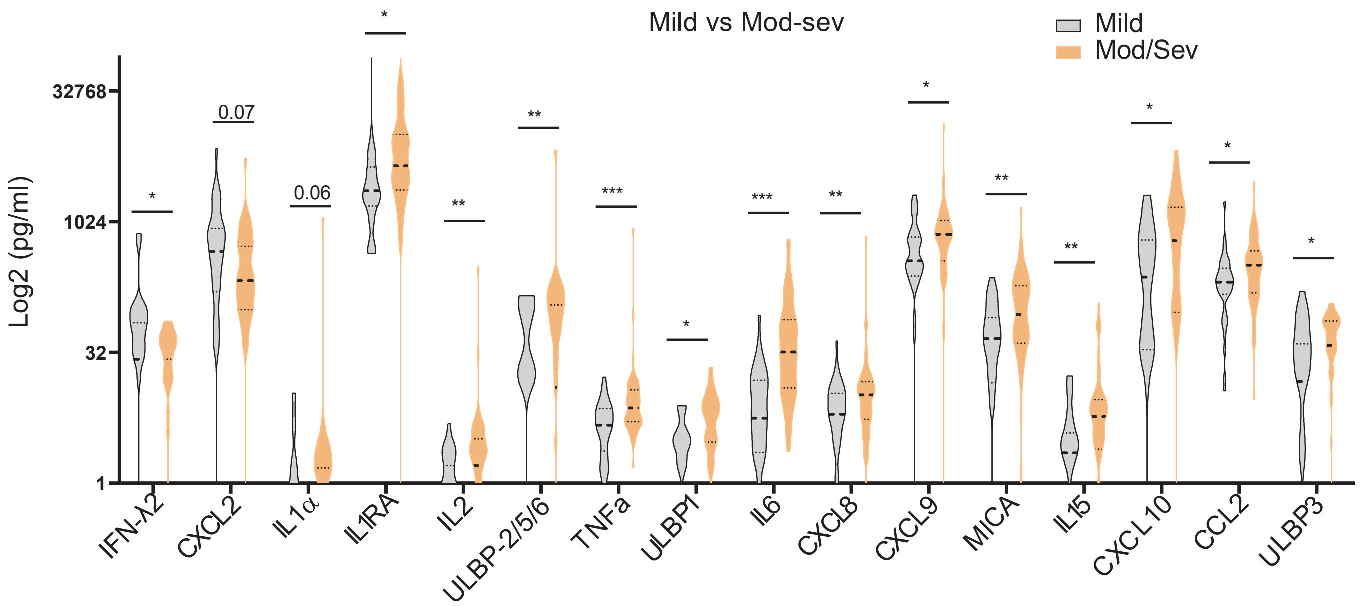

B)

	Mild vs Mod/Severe												
	Mild						Mod/Severe						
	N	Mean	SD	Median	P 25	P 75	N	Mean	SD	Median	P 25	C 75	p
IFN-λ2	35	70,5	155,9	26,8	0,0	70,4	51	14,5	21,6	0,0	0,0	26,8	0,015
CXCL2	35	830,8	1277,9	464,5	160,5	857,2	51	450,4	795,3	215,1	99,6	530,4	0,072
IL1β	35	0,7	2,0	0,0	0,0	0,4	51	23,3	160,6	0,1	0,0	1,5	0,061
IL1RA	35	4,3x10^{5}	1,7 x10^{6}	2,3 x10^{3}	1,6 x10^{3}	4,4 x10^{3}	51	3,3 x10^{7}	2,3 x10^{8}	4,5 x10^{3}	2,4 x10^{3}	1,0 x10^{4}	0,012
IL2	35	1,0	1,1	0,8	0,0	1,6	51	8,5	43,8	1,6	0,1	3,2	0,009
ULBP-2/5/6	35	15,2	37,9	0,0	0,0	0,0	51	186,2	954,0	12,7	0,0	112,6	0,002
TNFα	35	5,2	3,6	4,7	2,3	7,2	51	26,5	118,8	7,3	5,1	11,9	0,000
ULBP1	35	0,4	1,5	0,0	0,0	0,0	51	2,0	4,2	0,0	0,0	3,0	0,025
IL6	35	10,4	15,3	5,6	2,3	15,3	51	73,9	118,4	32,4	12,5	76,3	0,000
CXCL8	35	7,3	8,0	6,2	1,2	10,8	51	25,8	96,9	10,4	5,4	14,7	0,006
CXCL9	35	552,6	495,8	362,0	241,6	680,5	51	1037,2	1938,8	734,4	363,0	1058,1	0,014
MICA	35	58,3	57,3	46,0	14,3	80,4	51	155,3	225,1	87,3	40,8	187,6	0,001
IL15	35	3,5	4,5	2,2	0,2	3,8	51	11,7	21,5	5,9	2,5	9,2	0,002
CXCL10	35	423,3	538,5	236,4	34,4	629,7	51	1053,9	1345,2	617,3	92,6	1503,8	0,023
CCL2	35	259,2	290,3	206,5	149,5	298,3	51	410,5	451,7	323,8	155,2	472,3	0,025
ULBP3	35	28,3	38,2	14,8	0,0	40,2	51	42,7	33,9	38,7	8,9	73,7	0,041

SUPPLEMENTARY FIGURE 5

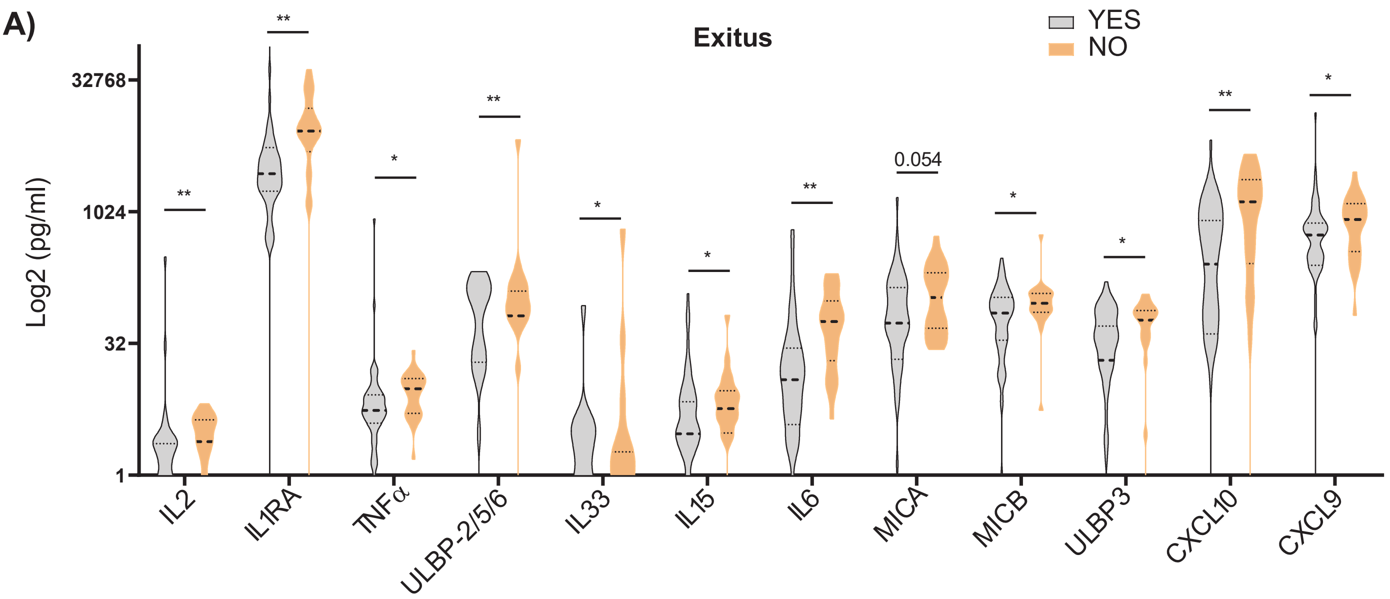

B)

	Exitus												
	No						Mean						
	N	Mean	SD	Median	P 25	P 75	N	Media	SD	Median	P 25	P 75	P
IL2	64	6,4	39,2	0,8	0,0	2,3	22	2,7	1,9	2,4	1,0	4,2	0,001
IL1RA	64	2,7 x10^7	2,1 x10^8	2,8 x10^3	1,7 x10^3	5,0 x10^3	22	1,2 x10^4	1,1 x10^4	8,6 x10^3	5,6 x10^3	1,4 x10^3	0,001
TNFα	64	20,7	106,4	5,5	3,8	8,1	22	9,6	5,5	9,7	5,1	12,7	0,018
ULBP-2/5/6	64	27,2	55,3	0,0	0,0	17,8	22	376,8	1447,0	66,1	0,0	121,1	0,001
IL33	64	1,9	10,8	0,0	0,0	0,6	22	32,0	138,3	0,4	0,0	1,8	0,026
IL15	64	8,1	18,3	2,9	0,8	6,8	22	9,2	13,9	5,8	3,1	9,2	0,053
IL6	64	40,8	105,8	12,1	4,1	28,0	22	69,3	59,0	57,0	22,5	86,0	0,000
MICA	64	105,9	198,3	54,1	20,3	135,1	22	144,9	126,7	106,9	51,2	187,6	0,024
MICB	64	78,0	60,1	70,2	33,9	106,6	22	111,3	104,7	91,9	73,2	118,0	0,054
ULBP3	64	32,0	35,2	20,5	0,0	50,3	22	51,1	36,2	59,1	20,0	74,9	0,032
CXCL10	64	581,3	975,0	247,3	40,9	805,9	22	1425,5	1328,0	1325,2	300,0	2227,7	0,002
CXCL9	64	817,5	1745,5	557,7	276,2	772,8	22	905,3	658,4	831,8	363,0	1238,6	0,034

SUPPLEMENTARY TABLE 1

	N	%
COVID19	86	53.4
NON-COV-RTI	27	16.7
HD	48	29.8
TOTAL	161	100

		HD	COVID19	NON-COV-RTI
		Average	Average	Average
Age		44 ± 12.5	71.2± 17.9	63.7± 22.6
Gender	Woman	47.9%	58.1%	59.35%
	Man	52.1%	41.9%	40.7%
Race	Caucasian		84.9%	88.9%
	Black		2.3%	0
	Hispanic		11.6%	11.1%
	Arabic		1.2%	0

	COVID19		NON-COV-RTI		p
	Average	SD	Average	SD	
Day since symptoms onset-hospitalization	6.14	5.39	8.04	13.8	0.202
Day since symptoms onset-extraction	6.12	5.39	8.36	13.6	0.472
Hospital stay(days)	12.24	12.9	6.5	10.7	0.705
Lymphocyte count	971.8	619.3	1285.2	837.0	0.014
Exitus	25.6%	-	14.8%	-	-

SUPPLEMENTARY TABLE 2

Classification	Parameters	Groups
1	Does not require oxygen therapy	Mild
2	Requires oxygen therapy at < 4 L/min	Moderate
3	Requires oxygen therapy at > 4 L/min	
4	Requires oxygen therapy at > 4 L/min but developed tachypnea (> 22 rpm or dyspnea at rest or minimal effort)	Severe
5	Requires no invasive support CPAP/BIPAP	
6	Mechanical ventilation PaO2/FiO2 > 150	
7	Mechanical ventilation PaO2/FiO2 ≤ 150	
8	Mechanical ventilation PaO2/FiO2 ≤ 150 and prone position/ECMO	

			Lymphocytes		
		N	Average	SD	p
Mild vs Moderate/Severe	Mild	35	1225.7	735.8	0.002
	Moderate/Severe	50	794.0	451.0	
Mild/Moderate vs Severe	Mild/Moderate	78	1015.4	624.4	0.009
	Severe	7	485.7	254.5	
Have improved after 7 days	No	60	998.3	681.6	0.774
	Yes	16	962.5	487.0	
Getting worse after 7 days	No	69	998.6	657.9	0.228
	Yes	7	914.3	501.4	
Exitus	No	63	1098.4	656.4	<0.001
	Yes	22	609.1	275.9	

		Mild		Moderate/Severe		p
		N	%	N	%	
Sex	Woman	21	60.0	29	56.9	0.826
	Man	14	40.0	22	43.1	
Race	Caucasian	27	77.1	46	90.2	0.322
	Black	1	2.9	1	2.0	
	Hispanic	6	17.1	4	7.8	
	Arabic	1	2.9	0	0.0	

	Mild		Moderate/Severe		p
	Average	SD	Average	SD	
Age	61.5	19.3	77.9	13.4	<0.001
Days since symptoms onset-hospitalization	8.00	7.10	4.86	3.31	0.051
Days since symptoms onset-extraction	7.91	7.12	4.88	3.34	0.072
Hospital stay (days)	5.7	7.8	17.1	13.6	<0.001

SUPPLEMENTARY TABLE 3

		Mild		Moderate/severe		p
		N	%	N	%	
Comorbidity (anyone)		20	57.1	49	96.1	<.001
Comorbidity	Chronic heart disease	1	2.9	14	27.5	0.003
	Hypertension	12	34.3	34	66.7	0.004
	Chronic lung disease	1	2.9	8	15.7	0.076
	Asthma	2	5.7	0	0.0	0.163
	Chronic kidney disease	3	8.6	7	13.7	0.518
	Diabetes mellitus	5	14.3	18	35.3	0.046
	Chronic neurologic disease	1	2.9	12	23.5	0.012
	Active solid neoplasm	1	2.9	0	0.0	0.0407
	Active hematologic neoplasm	1	2.9	1	2.0	1.000
	HIV/AIDS	0	0.0	0	0.0	
	Obesity (BMI ≥30 kg/m^2)	4	11.4	17	33.3	0.036
	Chronic inflammatory disease	3	8.6	2	3.9	0.393
	Dementia	3	8.6	18	35.3	0.006
	Smoking	0	0.0	2	4.0	0.509
	Others	0	0.0	6	11.8	0.077
Immunosuppressive agents (anyone)		4	11.4	2	3.9	0.219
Immunosuppressive agents	Systemic chemotherapy	2	5.7	0	0.0	0.163
	Systemic glucocorticoids	1	2.9	2	3.9	1.000
	Biological therapies	3	8.6	0	0.0	0.064
Intensive care		0	0.0	9	17.6	0.009
Barthel<60		5	14.3	27	52.9	<.001

SUPPLEMENTARY TABLE 4

		COVID-19		Non-COV-RTI		p
		N	%	N	%	
Comorbidity (anyone)		69	80.2	20	74.1	0.792
Comorbidity	Chronic heart disease	15	17.4	3	11.1	0.389
	Hypertension	46	53.5	14	51.9	0.744
	Chronic lung disease	9	10.5	7	25.9	0.104
	Asthma	2	2.3	1	3.7	0.548
	Chronickidney disease	10	11.6	4	14.8	0.699
	Diabetes mellitus	23	26.7	4	14.8	0.424
	Chronic neurologic disease	13	15.1	8	29.6	0.219
	Active solid neoplasm	1	1.2	3	11.1	0.005
	Active hematologic neoplasm	2	2.3	0	0.0	0.470
	HIV/AIDS	1	1.2	0	0.0	0.688
	Obesity(BMI ≥30 kg/m^2)	21	24.4	5	18.5	0.563
	Chronic inflammatory disease	5	5.8	3	11.1	0.142
	Dementia	21	24.4	7	25.9	0.854
	Smoking	2	2.4	2	7.4	0.053
	Others	6	7.0	3	11.1	0.915
Immunosuppressive agents(anyone)		6	7.0	3	11.1	0.695
Immunosuppressive agents	Systemic chemotherap	2	2.3	1	3.7	0.323
	Systemic glucocorticoids	3	3.5	1	3.7	0.520
	Biological therapies	3	3.5	2	7.4	0.263
Intensive care		9	10.5	0	0.0	0.217
Barthel<60		32	37.2	9	33.3	0.380

SUPPLEMENTARY TABLE 5

**Group 1**
Lymphocytes
Sex
Age
MICA
MICB
ULBP1
ULBP3
ULBP 2/5/6
IL12
IL25
IL2
IL15
IL6
IL1α
IL18
IL1RA
IL36B
IL33
IL31
IL7
IL10
CXCL8
CXCL10
CCL2
CXCL2
CXCL9
CCL8
IFN-λ2
IFN-λ3
INFα
INFβ
INFγ
TNFα
GzmA
GzmA activity (pM/min)
GzmB activity (pM/min)

**Group 2**
Sex
Age
Lymphocytes
T cells
$CD127^{-}CD25^{+}$ T^{reg}
NKT
NK
NK^{Dim}
NK^{Bright}
NK^{Dim} $NKG2A^{+}$
NK^{Dim} $NKp30^{+}$
NK^{Dim} $NKp46^{+}$
NK^{Dim} $NKG2D^{+}$
NK^{Dim} $CD57^{+}$
NK^{Dim} $NKG2A^{-}$ $CD57^{+}$ $NKG2C^{+}$
Activated $NK^{Dim}GzmB^{Brigh}TIM3^{+}$
Exhausted $NK^{Dim}GzmB^{Low}$ $TIM3^{+}$
Activated $NK^{Dim}GzmB^{Brigh}LAG3^{+}$
Exhausted $NK^{Dim}GzmB^{Low}LAG3^{+}$
Activated $NK^{Dim}GzmB^{Brigh}$ $PD1^{+}$
Exhausted $NK^{Dim}GzmB^{Low}PD1^{+}$
Mon
cMon
iMon
ncMon
cMon $TIM3^{+}$
cMon $LAG3^{+}$
cMon $PD1^{+}$
iMon $TIM3^{+}$
iMon $LAG3^{+}$
iMon $PD1^{+}$
ncMon $TIM3^{+}$
ncMon $LAG3^{+}$
ncMon PD1+
CD8 T cells
Activated $CD8^{+}$ $CD38^{+}$ $HLADR^{+}$
Activated $CD8^{+}CD38^{+}HLADR^{+}$ $TIM3^{+}$
Activated $CD8^{+}CD38^{+}HLADR^{+}$ $LAG3^{+}$
Activated $CD8^{+}CD38^{+}HLADR^{+}$ $PD1^{+}$
Exhausted $CD8^{+}(GzmB^{Low}$ $TIM3^{+})$
Exhausted $CD8^{+}(GzmB^{Low}$ $LAG3^{+})$
Exhausted $CD8^{+}(GzmB^{Low}$ $PD1^{+})$

SUPPLEMENTARY TABLE 6

COVID 19 vs HD		OR (95% CI)	p	AUC
Group 1 97,0%	IL15	1,42 (1,05-1,94)	0,024	0,996 (0,99-1,00); p< 0,001
	CXCL9	1,02 (1,00-1,04)	0,019	
	GzmA	1,26 (1,07-1,048)	0,005	
	GzmB activity (pM/min)	1,04 (1,00-1,07)	0,025	
Group 2 94,7%	nMon TIM3^{+}	0,819 (0,722-0,929)	0,002	0,990 (0,98-1,00); p< 0,001
	cMon TIM3^{+}	1,137 (1,035-1,249)	0,007	
	T^{reg}	1,695 (1,073-2,675)	0,024	
	Activated CD8T^{+}(CD38^{+}HLADR^{+}GzmB^{Bright})	9,807 (2,426-39,648)	0,001	

COVID 19 vs NON-COV-RTI		OR (95% CI)	p	AUC
Group 1 76,1%	CXCL10	1,002 (1,001-1,004)	0,006	0,70 (0,60-0,79); p= 0,002
Group 2 79,6%	nMon PD1^{+}	1,042 (1,016-1,069)	0,002	0,787 (0,690-0,885); p< 0,001
	Exhausted NK^{Dim}(GzmB^{Low} LAG3^{+})	1,054 (1,015-1,094)	0,006	
	T^{reg}	0,885 (0,780-1,004)	0,057	
	NK^{Dim}	1,088 (1,023-1,156)	0,007	

SUPPLEMENTARY TABLE 7

Mild vs Moderate/Severe		OR (95% CI)	p	AUC
Group 1 85.0%	Age	1,066 (1,020 -1,115)	0,005	0,93 (0,88 - 0,98); p< 0,001
	ULBP-2/5/6	1,017 (1,001 -1,033)	0,035	
	IL6	1,058 (1,018 -1,099)	0,004	
	IFN-λ2	0,949 (0,918 -0,980)	0,002	
Group 2 87,2%	Age	1,067 (1,022 -1,115)	0,003	0,91 (0,86 - 0,97); p< 0,001
	NKT	1,169 (1,019 -1,342)	0,026	
	Adaptive NK ($NK^{Dim}NKG2A^{-}$, $CD57^{+}$, $NKG2C^{+}$)	1,068 (1,009 -1,131)	0,024	
	Exhausted NK^{Dim}($GzmB^{Low}$ $LAG3^{+}$)	1,085 (1,001 -1,177)	0,048	
	Activated NK^{Dim} ($GzmB^{Bright}TIM3^{+}$)	0,889 (0,794 -0,994)	0,040	
	cMon $TIM3^{+}$	0,939 (0,889 -0,993)	0,026	
	cMon $LAG3^{+}$	0,966 (0,935 -0,998)	0,036	
	Exhausted $CD8^{+}$ T ($GzmB^{Low}$ $LAG3^{+}$)	1,095 (0,985 -1,216)	0,092	
	$CD38^{+}$ $HLADR^{+}$ $TIM3^{+}$	0,913 (0,848 -0,983)	0,016	
	$CD38^{+}HLADR^{+}$ $PD1^{+}$	1,083 (1,006 -1,166)	0,035	

COVID19 get worsen		OR (95% CI)	p	AUC
Group 1 83,1%	CXCL10	1,001 (1,000 -1,002)	0,020	0,79 (0,66- 0,93); p< 0,001
	CXCL9	1,001 (1,000 -1,002)	0,075	
	CCL8	0,995 (0,989 -1,001)	0,080	
	IFN α	1,038 (0,996 -1,082)	0,078	
	TNF α	0,931 (0,873 -0,994)	0,032	
	GzmB	1,013 (1,001 - 1,025)	0,033	
Group 2 80.5%	cMon $LAG3^{+}$	1,033 (1,009 - 1,057)	0,007	0,78 (0,66- 0,89);p=0,0 01
	NK^{Dim}	1,137 (1,021 -1,267)	0,020	

COVID19 Exitus		OR (95% CI)	p	AUC
Group 1 83,0%	Age	1,105 (1,038 -1,000)	0,002	0,90 (0,84 - 1,00); p< 0,001
	Lymphocytes	0,998 (0,996 -1,000)	0,018	
	CXCL10	1,001 (1,000 -1,002)	0,024	
	TNF α	0,911 (0,835 -0,995)	0,038	
Group 2 83,0%	Age	1,101 (1,035 -1,171)	0,002	0,90 (0,83 - 0,963); p< 0,001
	Lymphocytes	0,998 (0,996 -0,999)	0,009	
	iMon $TIM3^{+}$	0,946 (0,899 -0,996)	0,036	
	Exhausted $CD8^{+}$T ($GzmB^{Low}$ $PD1^{+}$)	1,588 (0,961 -2,624)	0,071	
